# Deubiquitylase YOD1 regulates CDK1 stability and drives triple-negative breast cancer tumorigenesis

**DOI:** 10.1186/s13046-023-02781-3

**Published:** 2023-09-04

**Authors:** Zhitao Han, Qi Jia, Jing Zhang, Miaomiao Chen, Lining Wang, Kai Tong, Weiwei He, Yajie Zhang, Weina Zhu, Ju Qin, Tao Wang, Tielong Liu, Yong Ma, Yuanming Chen, Siluo Zha, Chunlei Zhang

**Affiliations:** 1 School of Chinese Medicine, School of Integrated Chinese and Western Medicine, Nanjing University of Chinese Medicine, Nanjing, Jiangsu China; 2 Nanjing Hospital of Chinese Medicine, Nanjing University of Chinese Medicine, Nanjing, Jiangsu China; 3 Department of Orthopaedic Oncology, Shanghai Changzheng Hospital, The Second Affiliated Hospital of Naval Medical University, Shanghai, China; 4 Key Laboratory of Digital Technology in Medical Diagnostics of Zhejiang Province, Hangzhou, Zhejiang China; 5 Central Laboratory, Nanjing Hospital of Chinese Medicine, Nanjing University of Chinese Medicine, Nanjing, Jiangsu China; 6 Department of Biobank, Nanjing Hospital of Chinese Medicine, Nanjing University of Chinese Medicine, Nanjing, Jiangsu China; 7 Department of Orthopedics, Second Hospital of Anhui Medical University, Hefei, Anhui China; 8 Department of Orthopedics, Second Affiliated Hospital of Guangxi Medical University, 166 East Daxue Road, Nanning, 530000 Guangxi China; 9 Department of General Surgery, Shanghai Changzheng Hospital, The Second Affiliated Hospital of Naval Medical University, Shanghai, 200003 China; 10 Department of Orthopedics, Nanjing Hospital of Chinese Medicine Affiliated to Nanjing University of Chinese Medicine, 157 Daming Road, Nanjing, 210023 China

**Keywords:** Triple-negative breast cancer (TNBC), YOD1, CDK1, Ovarian tumor deubiquitinase, Ubiquitylation regulation

## Abstract

**Background:**

Accumulating evidence has demonstrated that aberrant expression of deubiquitinating enzymes is associated with the initiation and progression of Triple-negative breast cancer (TNBC). The publicly available TCGA database of breast cancer data was used to analyze the OTUD deubiquitinating family members that were correlated with survival of breast cancer and ovarian tumor domain-containing 2 (OTUD-2), or YOD1 was identified. The aim of present study was to assess YOD1 expression and function in human TNBC and then explored the underlying molecular events.

**Methods:**

We detected the expression of YOD1 in 32 TNBC and 44 NTNBC samples by qRT-PCR, Western blot and immunohistochemistry. Manipulation of YOD1 expression was assessed in vitro and in vivo for TNBC cell proliferation, migration, invasion, cell-cycle and drug resistance, using colony formation assay, transwell assay, CCK8 assay, TUNEL assay, flow cytometric analysis and xenograft tumor assay. Next, proteomic analysis, Western blot, proximity ligation assay, Immunoprecipitation, and Immunofluorescence were conducted to assess downstream targets.

**Results:**

It was found that YOD1 was significantly upregulated in TNBC tissues compared with non-triple-negative breast cancer (NTNBC), which was positively correlated with poor survival in TNBC patients. Knockdown of YOD1 effectively inhibited TNBC cell migration, proliferation, cell cycle and resistance to cisplatin and paclitaxel. Mechanistically, YOD1 promoted TNBC progression in a manner dependent on its catalytic activity through binding with CDK1, leading to de-polyubiquitylation of CDK1 and upregulation of CDK1 expression. In addition, YOD1 overexpression was found to be correlated with CDK1 overexpression in human TNBC specimens. Finally, in vivo study demonstrated that YOD1 knockdown or YOD1 inhibitor could inhibit CDK1 expression and suppress the growth and metastasis of TNBC tumors.

**Conclusion:**

Our study highlights that YOD1 functions as an oncogene in TNBC via binding to CDK1 and mediated its stability and oncogenic activity. Interfering with YOD1 expression or YOD1 inhibitor could suppress TNBC cells in vitro and in vivo, suggesting that YOD1 may prove to be a promising therapeutic target for TNBC.

**Supplementary Information:**

The online version contains supplementary material available at 10.1186/s13046-023-02781-3.

## Background

Breast cancer is a heterogeneous disease that can be subclassified as triple-negative breast cancer (TNBC) and non-triple-negative breast cancer (NTNBC). Clinically, TNBC is defined as the subtype tested negative for estrogen receptor (ER), progesterone receptor (PR) and human epidermal growth factor receptor 2 (HER2), accounting for 15–20% of all breast cancers [1, 2]. The main clinical characteristics of TNBC include easy recurrence, strong tumor invasiveness, a high degree of malignancy and high mortality [3, 4]. Due to the lack of effective targeted therapies other than chemotherapy, TNBC is the worst subtype of all types of breast cancer in terms of prognosis [5]. Therefore, the development of novel therapeutic targets for the precise treatment of TNBC is urgently needed.

Protein ubiquitination regulates numerous cellular processes in tumors, including protein degradation, cell signaling, growth cycle, apoptosis, and DNA damage response [6, 7]. This regulatory effect is achieved by the ability of ubiquitin (Ub) to form different Ub topologies via alternative conjugation signals [8]. However, protein ubiquitination can be reversed by deubiquitinating enzymes (DUBs) by removing Ub residues [9]. Being one of the DUB family members, ovarian-tumor related protease (OTU) recognizes specific Ub chains and represents the essential modulator of signaling cascades [10]. Numerous beneficial effects of OTU domain-containing proteins (OTUDs) have been reported to be involved in breast cancer development, proliferation, invasion, and metastasis. It was reported that OTUD1 could specifically inhibit transforming growth factor-beta (TGF-β) signaling in breast cancer metastasis by empowering SMAD7 [11]. OTUD3 could regulate PTEN stability and suppress human breast cancer progression [12]. Others reported that OTUD5-mediated YAP deubiquitination favored TNBC progression [13]. OTUD6A, OTUD6B and OTUD7B play an essential role in breast cancer occurrence and metastasis [14–17]. OTUD2, also known as YOD1, plays an essential role in tumorigenesis, such as cervical cancer [18]and ovarian tumor [19]. However, the role of YOD1 in human TNBC remains uninvestigated and the biological function and mechanism of YOD1 in TNBC remain to be explored.

In this study, we intend to clarify the function of YOD1 in the malignant biological behavior and chemotherapy resistance of TNBC, and further explore the potential molecular mechanism. It was found in our study that YOD1 functioned as an oncogene in TNBC via binding to CDK1 and mediating its stability. This new finding may help develop new targeted drugs for TNBC.

## Materials and methods

### Human tissues and datasets

A total of 32 primary TNBC and 44 primary NTNBC tissues were obtained from breast cancer patients who underwent mammectomy at the Department of Surgery in Shanghai Changzheng Hospital (Shanghai, China). The inclusion criteria were patients: (1) who underwent radical correction in breast cancer or breast-conserving surgery; (2) whose TNBC diagnosis was confirmed by pathology; (3) who had complete clinical data, pathological results and follow-up data; and (4) whose surgical pathological sections were well-preserved. Freshly resected samples were stored at − 80 °C or paraffin embedded. All procedures were conducted in accordance with the Declaration of Helsinki and approved by the Institutional Ethics Review Board of the said hospital. Written informed consent was obtained from all patients. In addition, TCGA public datasets were also analyzed.

### Cell lines

NTNBC cell lines (MCF10A, MCF7, T47D and ZR75-1), TNBC cell lines (MDA-MB-468, MDA-MB-231 and BT549), and a human embryonic kidney cell line (HEK293T) were obtained from the American Type Culture Collection (ATCC). They were cultured following the instructions from ATCC. HEK293T, MCF10A, MCF7, MDA-MB-468, MDA-MB-231 cells were cultured in DMEM with 10% fetal bovine serum (Gibco 11,765,054) + 1% Penicillin/streptomycin (Gibco 10,099,141) in a 37 °C incubator with 5% CO2. T47D, ZR75-1 and BT549 cells were cultured in RPMI-1640 with 10% FBS + 1% Penicillin/streptomycin at 37 °C with 5% CO2. All cell lines were authenticated by short tandem repeat analysis and tested negative for Mycoplasma contamination.

### Plasmid construction

YOD1 expression plasmids (pcDNA3.1-YOD1-FLAG), CDK1 expression plasmid (pcDNA3.1- CDK1-HA) and UB expression plasmids (pcDNA3.1-UB-His) were purchased from GENEWIZ. YOD1 was amplified by PCR from pcDNA3.1-YOD1-FLAG expression vector and subcloned into the pCDH-CMV-MCS-EF1-Puro vector for Lentivirus Package. The short hairpin (shRNA) oligos were designed by Sigma-Aldrich and synthesized by GENEWIZ. The shRNA was oligo annealed and cloned into the pLKO.1-Puro vector for Lentivirus Package. All truncations were amplified by PCR from pcDNA3.1-YOD1-FLAG and homologous recombination by ClonExpress® Ultra One Step Cloning Kit(Vazyme C115-01). The related Primers or shRNA sequences are listed in Supplementary Table 1.

### Antibodies and drugs

Antibodies and drugs used in this study were anti-YOD1 (PA5-35042), anti- CDK1 (MA5-11472), anti-beta Tubulin (MA5-11732), anti-P70S6K (PA5-28597), and anti-phospho-P70S6K (701,083) (Thermo Fisher Scientific, USA); anti-Ub (ab140601), anti-RB (ab181616), and anti- phospho-Rb (ab173289) (Abcam, USA); anti- Flag(66008-4-Ig), anti- HA (51064-2-AP) and anti-His (66005-1-Ig) (Proteintech, China); Cisplatin (HY-17,394), paclitaxel (HY-B0015), Ro-3306(HY-12,529), CDK1-IN-4(HY-151,408) and Ubiquitin Isopeptidase Inhibitor I, G5(HY-100,738) (MedChemExpress, USA); phosphatase inhibitors and protease inhibitor were obtained from Selleck (USA).

### Lentivirus package and infection

To generate stable cell lines expressing specific shRNAs or cDNAs, HEK293T cells were transfected with the appropriate lentiviral expression vector(pCDH vectors, pCDH-YOD1 or pLKO.1-sh-YOD1-1/2)and packaging plasmids (psPAX2 and pMD2.G) using Lipofectamine 3000 transfection reagent. Each virus-containing supernatant was collected 48 h after transfection and infected with the target cells (MDA-MB-231 and BT549) at 70% confluence, and 1 µg/mL puromycin was used to conduct drug-based selection for 1 week.

### RNA isolation and qRT-PCR

RNA was extracted from treated cells using TRIzol reagent (Invitrogen 15596-026) and reverse transcribed to cDNA using a PrimeScript RT Reagent Kit with gDNA Eraser (TaKaRa RR037A) following the manufacturer’s recommended protocols. Primers were designed from information available on the PrimerBank website. qRT-PCR was performed using SYBR Premix Ex Taq (TaKaRa RR420A) in an ABI 7900HT Fast Real-Time PCR System (Applied Biosystems). qRT-PCR data for mRNA expression levels were shown relative to the expression levels of the reference gene. The formula 2(−ΔCt) was used to calculate the relative expression values. The gene-specific primers used are listed in Supplementary Table 1.

### Western blot

Proteins were extracted by RIPA containing the phosphatase inhibitor and protease inhibitor. The protein concentration was determined by BCA kit (Beyotime P0012). 10–12% polyacrylamide gels were used to separate protein. Then the protein was transferred to PVDF membranes. After blocking in 5% BSA, the membranes were incubated with the indicated primary antibodies at 4℃ overnight, followed by addition of the corresponding HRP conjugated secondary antibodies to the membranes.

### Immunohistochemical (IHC) staining

Tumor tissues were fixed with 4% paraformaldehyde, dehydrated through a graded series of ethanol, paraffin embedded and sliced into 5-µm sections. The paraffin-embedded tissue sections were deparaffinized at 60 °C for 20 min, cleared in xylene, and rehydrated in a graded alcohol series. IHC staining was performed as described previously with anti-YOD1 (1:100) primary antibodies [19]. The IHC results were independently interpretated by two pathologists who were blinded to the clinicopathologic information. The percentage of the positively stained area (0 = negative, 1 < 10%, 2 = 10–50%, and 3 > 50%) was recorded for each sample. Low and high expressions were defined according to the median immunoreactivity score.

### Immunofluorescence

Cells were fixed with paraformaldehyde for 15 min and permeabilized with Triton X-100 for 20 min at room temperature. Cells or tissue sections then were blocked with 1% BSA solution for 1 h at room temperature and incubated with primary antibody (1:50) at 4 °C overnight. The secondary antibody (1:400) was diluted in PBS and incubated at room temperature for 1 h. The slides were counterstained with DAPI. Finally, cell nuclei were stained with DAPI(Servicebio G1012-10ML), and the fluorescence signal was captured using the fluorescence microscope (IX71; Olympus Corporation, Tokyo, Japan).

### Immunoprecipitation

To immunoprecipitate exogenously expressed and endogenous proteins, cells were lysed in NP-40 Lysis Buffer containing the protease inhibitor, and incubated with primary antibodies or control IgG in a rotating incubator overnight at 4 °C, followed by incubation with protein A/G magnetic beads (Bimake B23201) for additional 4 h at 4 °C. The immunoprecipitates were washed three times with PBST Buffer and analyzed by immunoblotting.

### GST pull-down

The plasmids for GST-YOD1 and His-CDK1 were transfected into E. coli BL21 (C504-02, vazyme) and purified by affinity chromatography using an AKTA protein purification system. The recombinant proteins were prepared as described previously [20, 21]. For GST pulldown, Glutathione Sepharose 4B beads were saturated with GST or GST-YOD1 for 1 h at 4˚C, followed by extensive washing. Subsequently, the His-CDK1 was incubated with the bead-bound GST-protein in assay buffer complemented with 0,5% Triton X100 for 2 h at 4˚C. Again, beads were washed extensively. Pulldowns were analyzed by SDS-PAGE and Coomassie Staining or Western Blotting.

### LC-MS/MS assay

293T cells were transfected with pcDNA3.1-YOD1-FLAG or pcDNA3.1- and harvested after 48 h. Whole cell protein was extracted using NP-40 Lysis Buffer supplemented with the protease inhibitor and immunoprecipitated with Anti-Flag Affinity Gel. The immunoprecipitates were washed three times with PBST Buffer and separated by SDS-PAGE. The bands of pcDNA3.1-YOD1-FLAG group or pcDNA3.1- group were cut from the gel. The sliced gel samples were analyzed by liquid chromatography tandem mass spectrometry (LC-MS/MS) performed in Shanghai Applied Protein Technology (APTBIO, China).

### Proximity ligation assay

Situ protein interactions in MDA-MB-231 or BT549 were detected by proximity ligation assay. Cells were seeded on slides and permitted to attach overnight, and fixed with 4% paraformaldehyde and permeabilized with 0.3% Triton X-100 in PBS. The cells were processed using the manufacturers’ protocol of the Duolink In Situ Red Starter Kit (Sigma-Aldrich, DUO92101) where the cells were probed for either rabbit anti-YOD1 or mouse anti-CDK1 or both followed by ligation and amplification process. Finally, cell nuclei were stained with DAPI, and the PLA signal was captured using the fluorescence microscope (IX71; Olympus Corporation, Tokyo, Japan).

### Colony formation assay

MDA-MB-231 and BT549 stably transfected cells were seeded in a 6-well plate. The culture medium was refreshed every other day. Cells were cultured for 7 days. Then the clones were fixed with 4% paraformaldehyde, stained with 0.1% crystal violet, and counted manually.

### Transwell assay

Migration and invasion assays were performed by using transwell chambers presence of Matrigel (Corning 354,480). Briefly, MDA-MB-231 and BT549 stable transfected cells (1 × 105 cells/well) were seeded into the upper chamber with serum-free medium, and 600 µl 20% FBS was added to the lower chamber of the 24-well plate. After 24-h incubation, the cells on the upper surface of the filter were completely removed by wiping with a cotton swab. Then, the filters were fixed with 4% paraformaldehyde and stained with crystal violet (Beyotime C0121). The cells in five randomly selected visual fields were counted under a microscope (DM500; Leica, Wetzlar, Germany) at 100× magnification.

### CCK8 assay

Cells were seeded in a 96-well plate at a density of 5000 cells per well. After 24-h incubation, the cells were treated with the indicated agents for 24 h, and then 10 µl Cell Counting Kit-8 (CCK-8) solution was added to each well for 2 ~ 4 h (HY-K0301, MedChemExpress, China). The optical absorbance (AD) at 450 nm was measured using an ELx800 microplate reader (BioTek Instruments Inc., USA).

### Terminal dexynucleotidyl transferase-mediated dUTP nick end labeling (TUNEL) assay

MDA-MB-231 and BT549 cells were cultured in 12-well plates and treated with cisplatin or paclitaxel for 24 h, washed with cold PBS, and fixed with 4% paraformaldehyde. TUNEL assay was performed using the TMR (red) Tunel Cell Apoptosis Detection Kit(Servicebio G1502-50T)according to the manufacturer’s instructions. Finally, cell nuclei were stained with DAPI, and the fluorescence signal was captured using the fluorescence microscope (IX71; Olympus Corporation, Tokyo, Japan).

### Flow cytometric analysis

Cell-cycle analysis was performed by propidium iodide (PI; Sigma-Aldrich 537,060) staining to quantify the sub-G1, S and G2 population, knowing that it can reflect the extent of cell cycle. Briefly, 1 × 10^5^ cells were seeded in a 6-well plate. After 24 h, cells were treated with the indicated agents for 72 h, fixed with 70% ethanol and stained with PI (50 µg/mL). The stained cells were tested by SP6800 Spectral Cell Analyzer (Sony) and analyzed by using the FlowJo software. For apoptosis assay, cells were treated with the indicated compounds and stained with 5ul FITC-Annexin V (BD Biosciences 556,419) and 5ul PI (BD Biosciences 556,463). After 15 min, the stained cells were analyzed by flow cytometry.

### Ubiquitination assay

Cells were transfected as indicated. To detect CDK1 ubiquitinated with His-conjugated Ub or endogenously ubiquitinated proteins, cells were lysed in NP-40 Lysis Buffer and immunoprecipitated by CDK1 primary antibodies. The immunoprecipitates were detected by immunoblotting and normalized by CDK1.

### In vivo tumor studies

BALB/c nude mice were purchases from the Animal Husbandry Center of Shanghai Institute of Cell Biology, Academia Sinica (Shanghai, China). For subcutaneous xenograft assay, female BALB/c nude mice aged 8 weeks were subjected to subcutaneous implantation into the dorsolateral side of the flank region for the MDA-MB-231 cell ( 5 × 106/100µl PBS). Tumor size was measured every three days using a vernier caliper. The mean tumor volume was calculated using the following equation: volume = 0.52 × length × Width. Xenograft tumors were removed, imaged, and weighed after euthanizing the animals at the end of the study.

In vivo distant metastasis was monitored by bioluminescent imaging. Briefly, MDA-MB-231 cells stably expressing luciferase by lentivirus transfection were injected into mice through the tail vein. When the tumors were detected by bioluminescent imaging, mice were treated with G5 (20 mg/kg; i.p.) every other day for 20 days. D-luciferinpotassium salt (AOK Chem, Shanghai) was injected intraperitoneally into the anesthetized mice at a dose of 150 mg/kg. After 10 min, the mice were placed into the IVIS Imaging System. Imagines were recorded with an exposure time of 2 min. Bioluminescence was assayed and photons per second were quantified using software (Living Image 3.2, Caliper).

Animal experiments were performed according to guidelines approved by the Institutional Animal Care and Use Committee, and performed in accordance with the guidelines for animal experimentation of the hospital and approved by the Committee for Animal Experimentation (No. 2020SY118, July 13th, 2020).

### Statistical analysis

Statistical analyses were performed using GraphPad Prism 7.0. Survival curves were plotted using the Kaplan-Meier method and compared using log-rank tests. Other data are presented as the mean ± standard error of the mean (SEM). Statistics of the mean value between groups was assessed using Student t test or ANOVA, assuming double-sided independent variance. For evaluating differences between groups, the Fisher exact test or Pearson chi-square test was used for categorical variables assuming double-sided independent variance. P < 0.05 was considered significant. ** means P < 0.01, ***means P < 0.001, and **** means P < 0.0001.

## Results

### YOD1 upregulation is inversely correlated with poor prognosis in TNBC

**Fig. 1:**
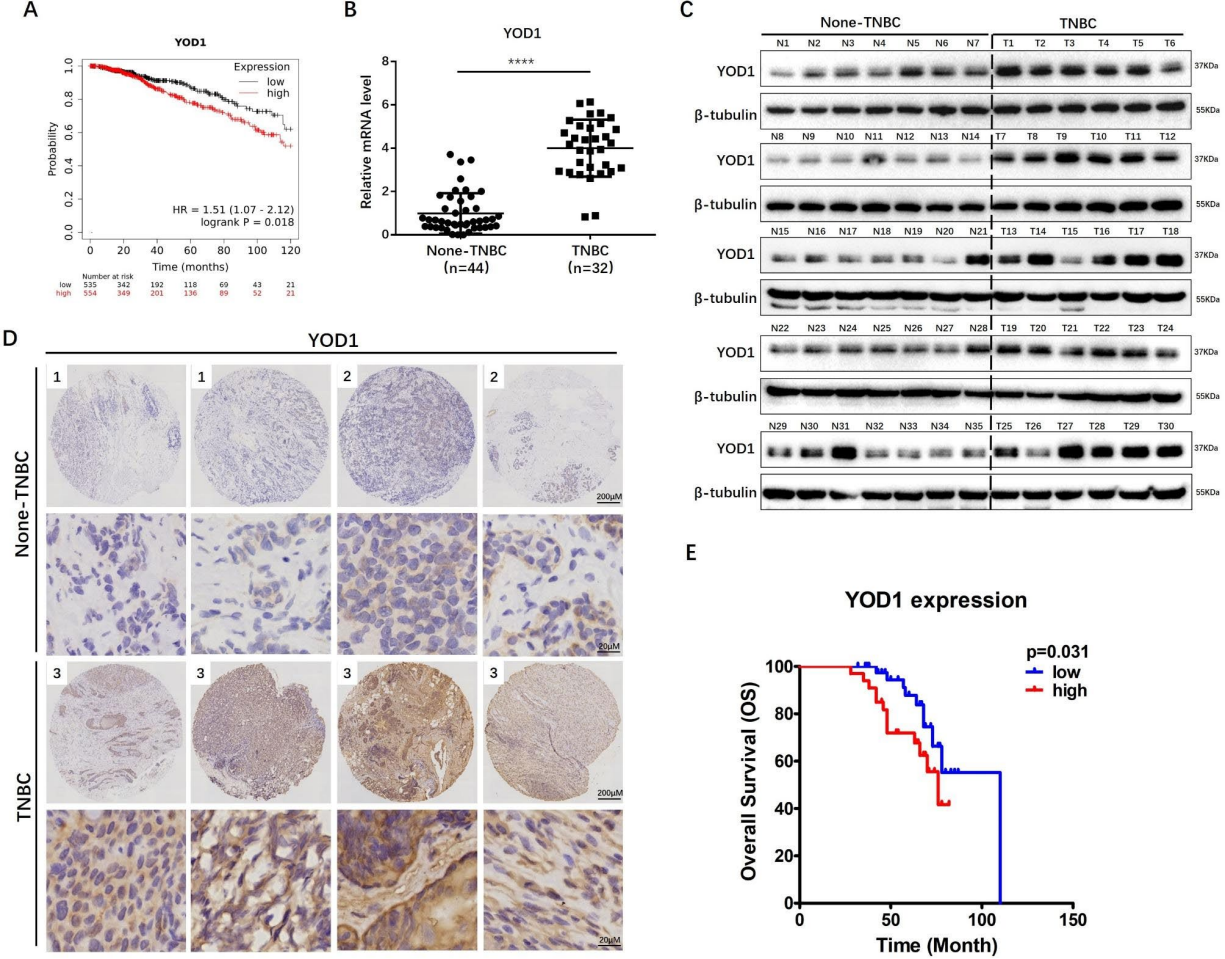
YOD1 is significantly upregulated in TNBC and inversely correlated with poor prognosis. **A**, Kaplan-Meier curve of BC showing that higher YOD1 expression was associated with poor prognoses based on TCGA database. **B**, YOD1 mRNA levels in 32 TNBC tissues and 44 none-TNBC tissues by qRT-PCR. **C**, YOD1 protein expression was quantified in primary TNBC tissues and none-TNBC tissues by Western blot. **D**, Five-micrometer (5-µm) sections were analyzed by IHC using anti-YOD1 antibodies. The percentage of positively stained area (1 < 10%, 2 = 10–50%, 3 > 50%) were recorded for each sample. **E**, Kaplan-Meier curve showing that the correlation between YOD1 expression levels and overall overall survival in breast cancer patients. Scale bars, 200 μm or 20 μm. Data are mean ± SEM. Two-tailed Student t test. ***, P < 0.001

Firstly, we used the publicly available TCGA database of breast cancer data to analyze the OTUD family members that were correlated with survival. Kaplan-Meier curves showed that OTUD2 (YOD1), OTUD3, OTUD6A and OTUD6B were inversely correlated across TNBC samples, while OTUD7A and OTUD7B were positively correlated with survival prognosis in TNBC (Fig. 1A & SFig.1 A). Then, we detected the expression of YOD1 in 32 TNBC and 44 NTNBC samples from our hospital by qRT-PCR and Western blot, and found that YOD1 was significantly up-regulated in TNBC tissues as compared with that in NTNBC tissues (Fig. 1B, C & Fig.S1B). The in-situ expression of YOD1 protein by IHC staining was detected in all samples and the staining intensity was recorded for each sample (Fig. 1D < 10%, 2 = 10–50%, 3 > 50%). It was found that higher YOD1 expression level (scored at 3) was related to a lower overall survival rate (Fig. 1E, p = 0.031) and higher proportion of TNBC patients (p < 0.001, Table 1). In addition, the expression level of YOD1 was positively correlated with the disease stage (p = 0.002, Supplement Table 2). All these findings demonstrated that patients with a higher expression level of YOD1 were prone to poorer survival outcomes, and YOD1 was significantly upregulated in TNBC.

**Table 1:** Correlation of YOD1 expression with clinical features of patients with breast cancer

Features	YOD1 expression level		χ²	P
	high	low		
Age				
< 50	15	25	1.205	0.355
≥ 50	18	18		
TNM stage				
I-II	13	33	10.902	0.002
III-IV	20	10		
Pathologic diagnosis				
NTNBC	10	34	18.215	< 0.001
TNBC	23	9		

### YOD1 overexpression promotes TNBC cell proliferation, migration and invasion

**Fig. 2:**
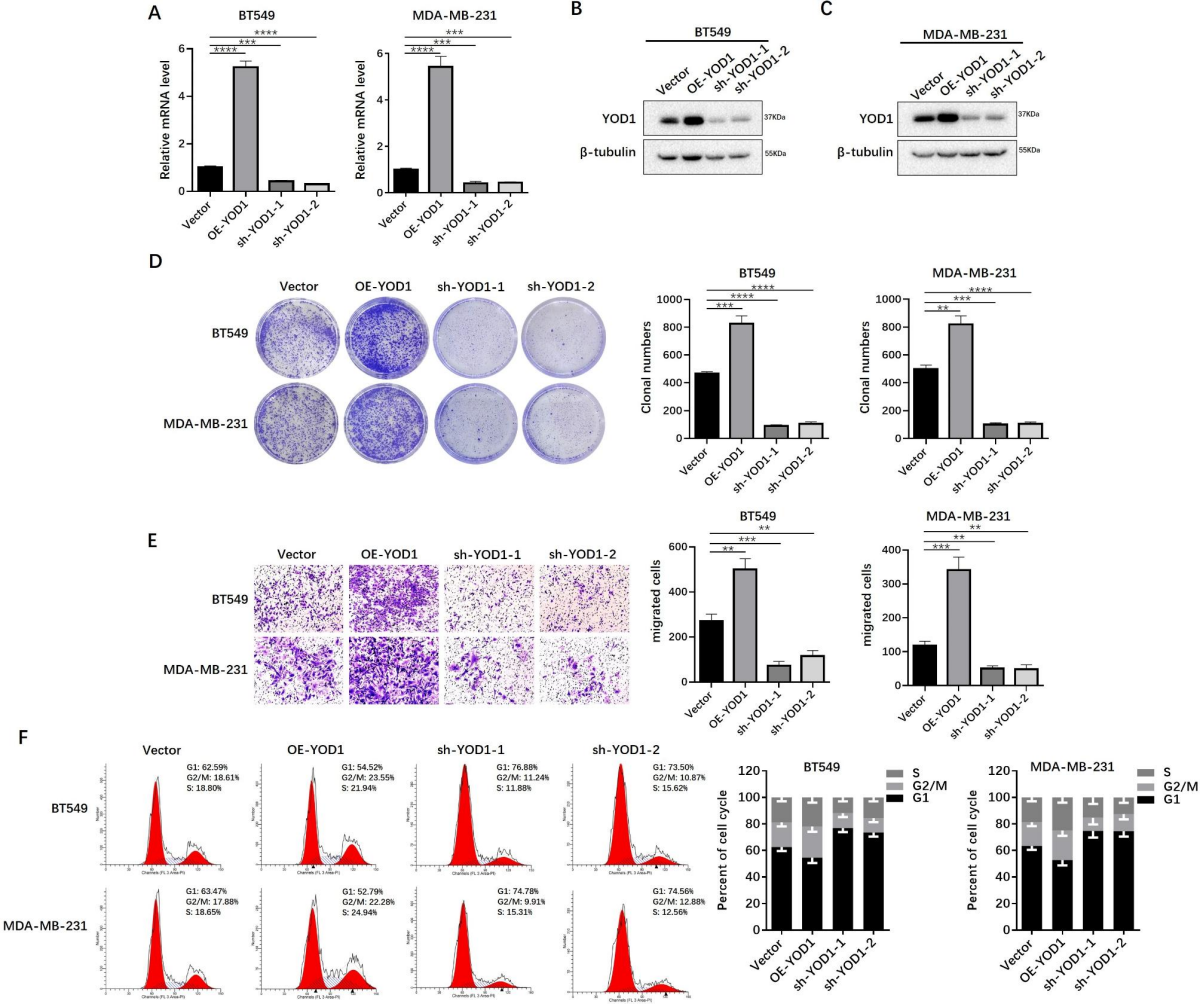
YOD1 drives TNBC cell proliferation, migration and invasion. **A-C**, Validated the mRNA and protein expression of YOD1 gain and loss in BT549 and MDA-MB-231 cells by qRT-PCR and western blotting assays. **D**, Colony formation assay were performed and number of colony formation assessed of the indicated TNBC cells which over-expression or Knockdown YOD1. **E**, Transwell assays were performed to evaluate the effects of YOD1 on the migration of the indicated TNBC cells. Representative images and quantification of relative migrated cells are shown. **F**, Cell cycle was evaluated by the effects of YOD1 function using flow cytometry in the indicated TNBC cells. Evaluated YOD1 significantly promoted cell population at S/G2 phase. Statistical analysis was performed using one-way ANOVA test. Mean ± SEM (**P < 0.01, ***P < 0.001, and****P < 0.0001)

The mRNA and protein expressions of YOD1 in multiple NTNBC and TNBC cell lines were detected, and the results showed that YOD1 expression in TNBC cell lines was significantly higher than that in NTNBC (P < 0.01) (Fig. S2 A-C). To determine the function of YOD1 in TNBC, we stably overexpressed or knocked down YOD1 in BT-549 and MDA-MB-231 cells via lentiviral infection, as confirmed by qRT- PCR (Fig. 2A) and Western blotting (Fig. 2B C). We next investigated the oncogenic property of YOD1 in BT-549 and MDA-MB-231 cell lines using soft agar colony formation assay. It was found that YOD1 Knockdown inhibited the clone formation ability, while YOD1 overexpression promoted the oncogenic capacity in both cell lines (Fig. 2D). Transwell invasion assays were performed to test the effect of YOD1 on TNBC cell migration and invasion. The results showed that YOD1 knockdown suppressed the migration and invasion abilities of BT-549 and MDA-MB-231 cell lines, while YOD1 overexpression markedly promoted the migration and invasiveness in both two cells (Fig. 2E). Knowing that dysregulation of the cell cycle is an important reason for tumor proliferation [22], we then used flow cytometry to determine whether cell-cycle arrest was engaged in the downregulation of proliferation when YOD1 level was decreased or increased in BT-549 and MDA-MB-231. It was found that YOD1 knockdown by siRNA led to conspicuous S–G2 phase arrest and S phase reduction in the cell cycle, while YOD1 overexpression promoted cell cycle progression (Fig. 2F).

### Overexpression of YOD1 is associated with breast cancer cell drug resistance

**Fig. 3:**
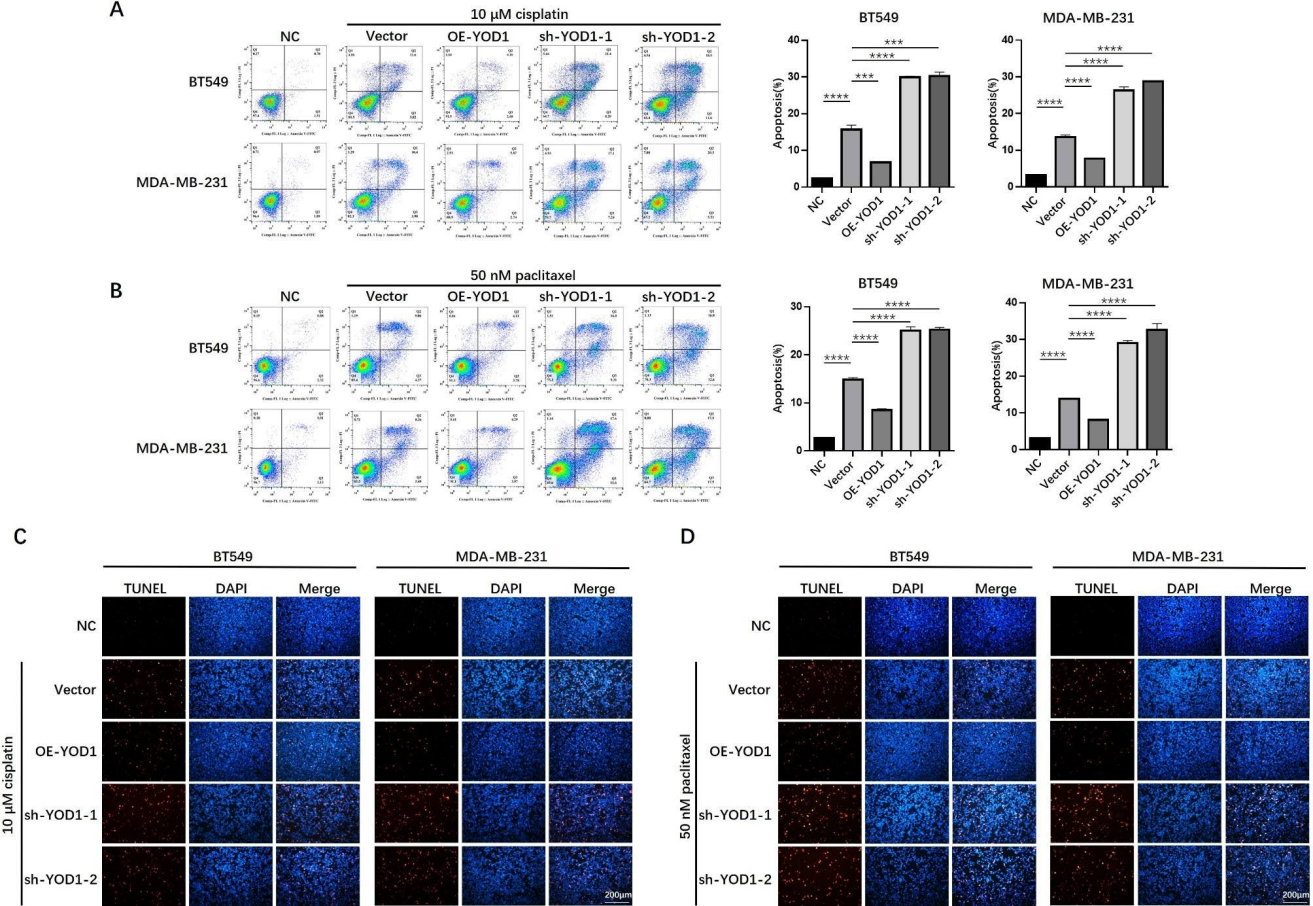
Overexpression of YOD1 induced cisplatin and paclitaxel resistance, and YOD1 knockdown significantly increased drug sensitivity in BT549 and MDA-MB-231 cells. **A-B**, Flow cytometry assays were performed to evaluate the effects of YOD1 gain and loss of function on the 10 µM cisplatin or 50 nM paclitaxel induced apoptosis of the indicated TNBC cells. **C-D**, TUNEL staining assays were conducted to evaluate the effects of YOD1 gain and loss of function on the 10 µM cisplatin or 50 nM paclitaxel induced apoptosis of the indicated TNBC cells. Data are mean ± SEM. Scale bars, 200 μm. one-way ANOVA test. (***P < 0.001, and****P < 0.0001)

Paclitaxel and cisplatin are representative chemotherapy agents for some advanced or recurrent tumors, including TNBC due to the lack of effective targeted therapies [23]. We detected apoptosis by flow cytometry (annexin-V) to verify whether YOD1 was involved in the drug resistance when YOD1 level decreased or increased in BT-549 and MDA-MB-231. Subsequently, we treated TNBC cells in a DMSO control group and a paclitaxel or cisplatin group for 24 h. The results showed that TNBC cell lines overexpressing YOD1 were more resistant to cisplatin or paclitaxel and decreased apoptosis, while YOD1 knockdown increased the sensitivity to cisplatin or paclitaxel, resulting in apoptosis (Fig. 3A and B). Furthermore, YOD1 overexpression and knockdown in normal breast cell line MCF-10 A were constructed. Flow cytometry showed that YOD1 overexpression or knockdown affect the apoptosis of MCF-10 A, but the degrees were significantly lower than TNBC (Supplementary Fig. 3A&3B). Next, we performed a TUNEL assay to detect apoptosis in BT-549 and MDA-MB-231. As expected, cisplatin or paclitaxel-induced TNBC cell apoptosis was enhanced by YOD1 knockdown and reduced by YOD1 overexpression (Fig. 3C and D).

### YOD1 interacts directly with CDK1

**Fig. 4:**
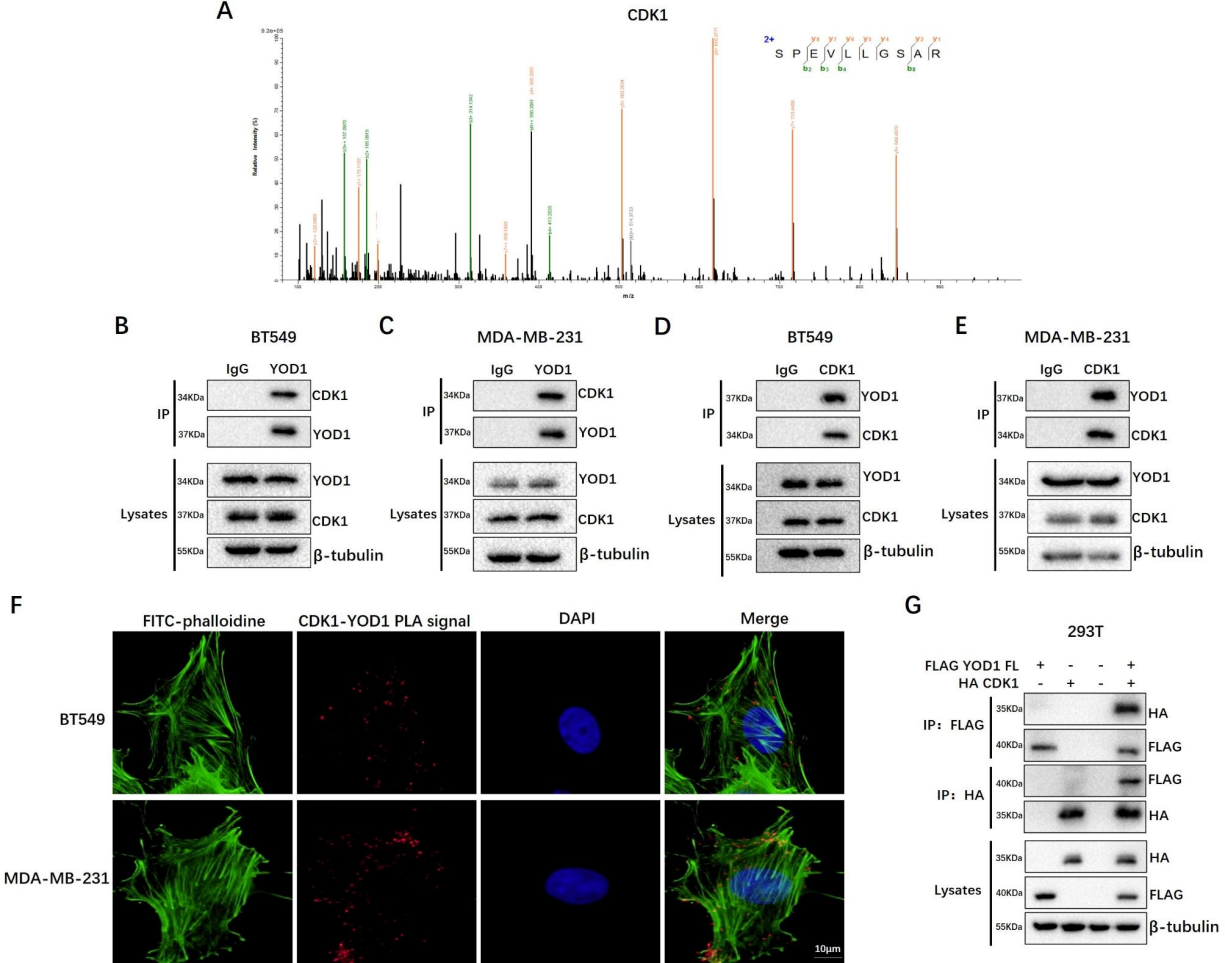
YOD1 interacts directly with CDK1 in TNBC cells. **A**, Specific peptide segments of CDK1 using mass spectrometry. **B-E**, Immunoprecipitation experiments were performed to evaluate endogenous interaction of YOD1 and CDK1 of the indicated TNBC cells. BT549 and MDA-MB-231 cell lysates were subjected to immunoprecipitation with control IgG, anti-YOD1 or anti-CKD1 antibody. The immunoprecipitates were then detected using the indicated antibodies. **F**, Detected YOD1/CDK1 relationships in indicated TNBC cells by in situ proximity ligation assay(PLA). PLA signal (Red fluorescence) was appeared in situ of BT549 and MDA-MB-231 cell. **G**, Flag-YOD1 and HA-tagged CDK1 were co-transfected into 293T cells. The cell lysates were subjected to immunoprecipitation with anti-Flag or anti-HA antibodies, and the interactions of exogenous YOD1 and CDK1 be detected. Scale bars, 10 μm

As described above, YOD1 played a vital role in TNBC progression. To further gain insights into the molecular mechanism, LC-MS/MS assay was performed to identify potential proteins binding to YOD1 (Fig. S4A&4B). Interestingly, CDK1 was identified as an interacting target with high specificity to YOD1 as shown by MS analysis (Fig. 4A). To further evaluate protein interactions, we analyzed protein interactions in cells using immunoprecipitation, proximity ligation (PLA) and bimolecular fluorescence complementation (BiFC). When YOD1 as the precipitated protein was separated with protein A/G magnetic beads and immunoblotted with the indicated antibody, CDK1 protein was detected in BT-549 and MDA-MB-231(Fig. 4B C). Similarly, CKD1 was also detected in YOD1 protein as the precipitated protein by SDS-PAGE in both cell lines (Fig. 4D and E). To clarify the relationship between YOD1 and CDK1, we performed a PLA analysis in which the presence of positivity as a red fluorescent signal indicates the in situ proximity between the two proteins. An in situ proximity of YOD1 and CDK1 was observed in both cell lines (Fig. 4F), which was also confirmed by BiFC assays in HEK293T (Fig. S5A). Furthermore, FLAG-tagged YOD1 or HA‐tagged CDK1 was co‐transfected into 293T cells to detect interactions of exogenous YOD1 and CDK1 by immunoprecipitation. The result showed that FLAG was detectable when HA precipitated, and HA was also detectable when FLAG precipitated, demonstrating a direct interaction between YOD1 and CDK1(Fig. 4G). Meantime, recombinant GST-YOD1 and HIS-CDK1 proteins were purified, and the GST pulldown results further excluded the direct interaction between YOD1 and CDK1 ( Fig. S6).

### CDK1 binds to the C-terminal znf domain of YOD1

**Fig. 5:**
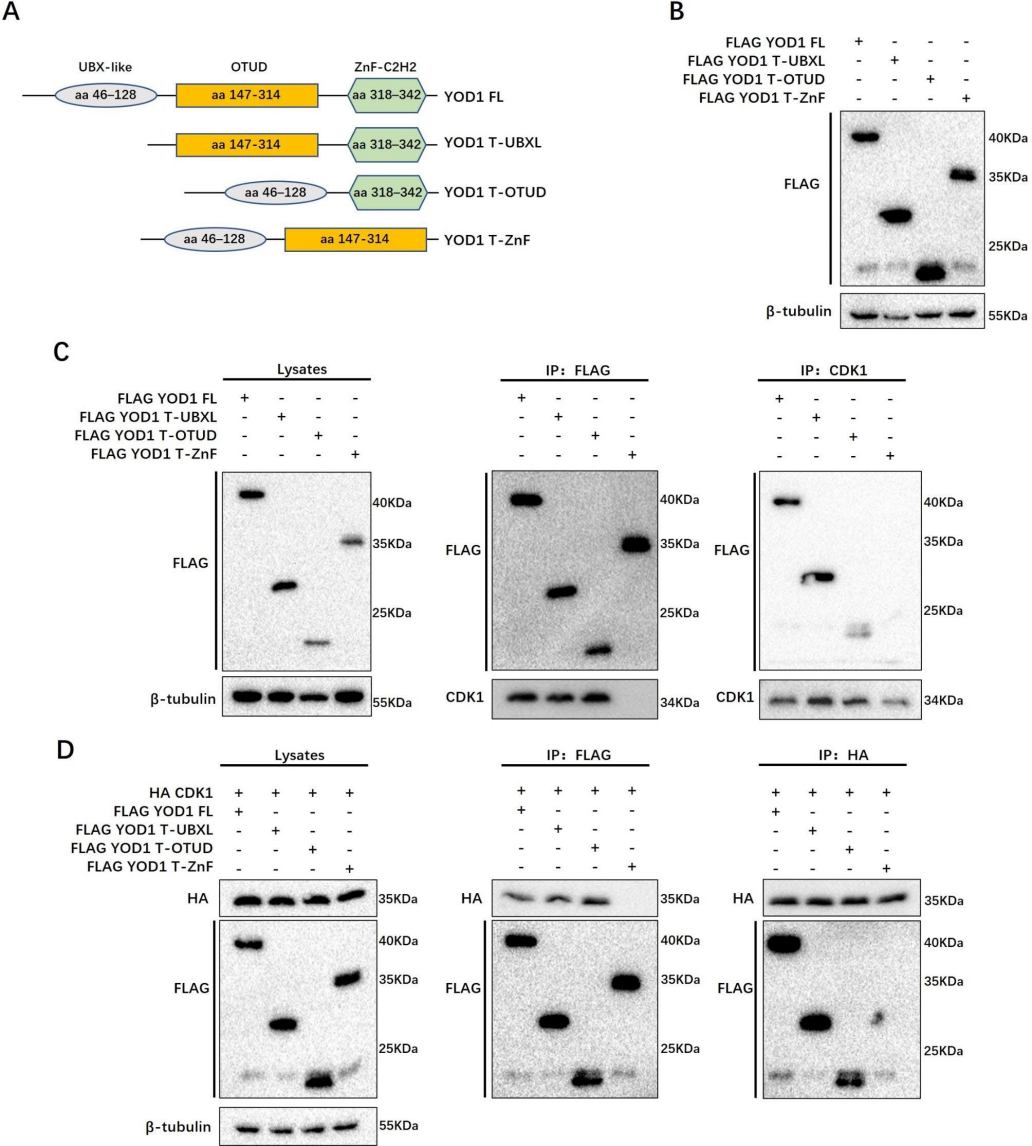
CDK1 binds to the C-terminal Znf domain of YOD1. **A**, Schematic of YOD1 structures and three different truncated constructs tagged with FLAG (UBXL, OTUD, Znf) were designed. **B**, The expression of YOD1 and specific truncations tagged with FLAG were verified by western blotting using the indicated anti-Flag antibody. **C**, Flag-YOD1 or its three truncated constructs were transfected respectively into 293T cells. The cell lysates were subjected to immunoprecipitation with indicated anti-Flag or anti-CDK1 antibodies, then western blot was performed to detected coprecipitation phenomena. **D**, HA-tagged CDK1 or Flag-tagged YOD1 and its truncated constructs were co-transfected into 293T cells. The cell lysates were subjected to immunoprecipitation with anti-Flag or anti-HA antibodies

Subsequently, we further explored the CDK1 binding site on YOD1 structural domain. Knowing that YOD1 comprises three principal domains: an N-terminal UBX domain, a central otubain domain, and a C-terminal C2H2-type Zinc finger (Znf) domain [24], we created truncated YOD1 constructs were created and designated YOD1-T-UBXL (no N-terminal UBX domain), YOD1-T-OTUD (no central otubain domain) and YOD1-T-ZnF (no C-terminal domains) constructs, which were tagged with the FLAG (Fig. 5A).

The expression vector of each YOD1 truncation mutant was transfected into 293T cells and the expression of specific truncations was verified by Western blotting using an anti-Flag antibody (Fig. 5B). Next, we expressed full-length FLAG-YOD1 and different truncation mutants in HEK293 cells and co-immunoprecipitations using the anti-FLAG antibodies or anti-CDK1 antibodies confirmed that CDK1 bound to the Znf domain of YOD1, but not to the N-terminal UBX region and central otubain region of YOD1 (Fig. 5C). We further co-expressed HA-tagged CDK1 with various Flag-tagged YOD1 mutants in HEK293 cells to identify which domain YOD1 bound to the CDK1 by performing reciprocal co-immunoprecipitation. Congruent with the above results, CDK1 specifically bound to the C-terminal Znf domain of YOD1 rather than the N-terminal UBX region and central otubain region of YOD1 (Fig. 5D). Then, we further confirmed these findings using BiFC (Fig. S5B).

### YOD1 deubiquitylates CDK1 and maintains CDK1 stability

**Fig. 6:**
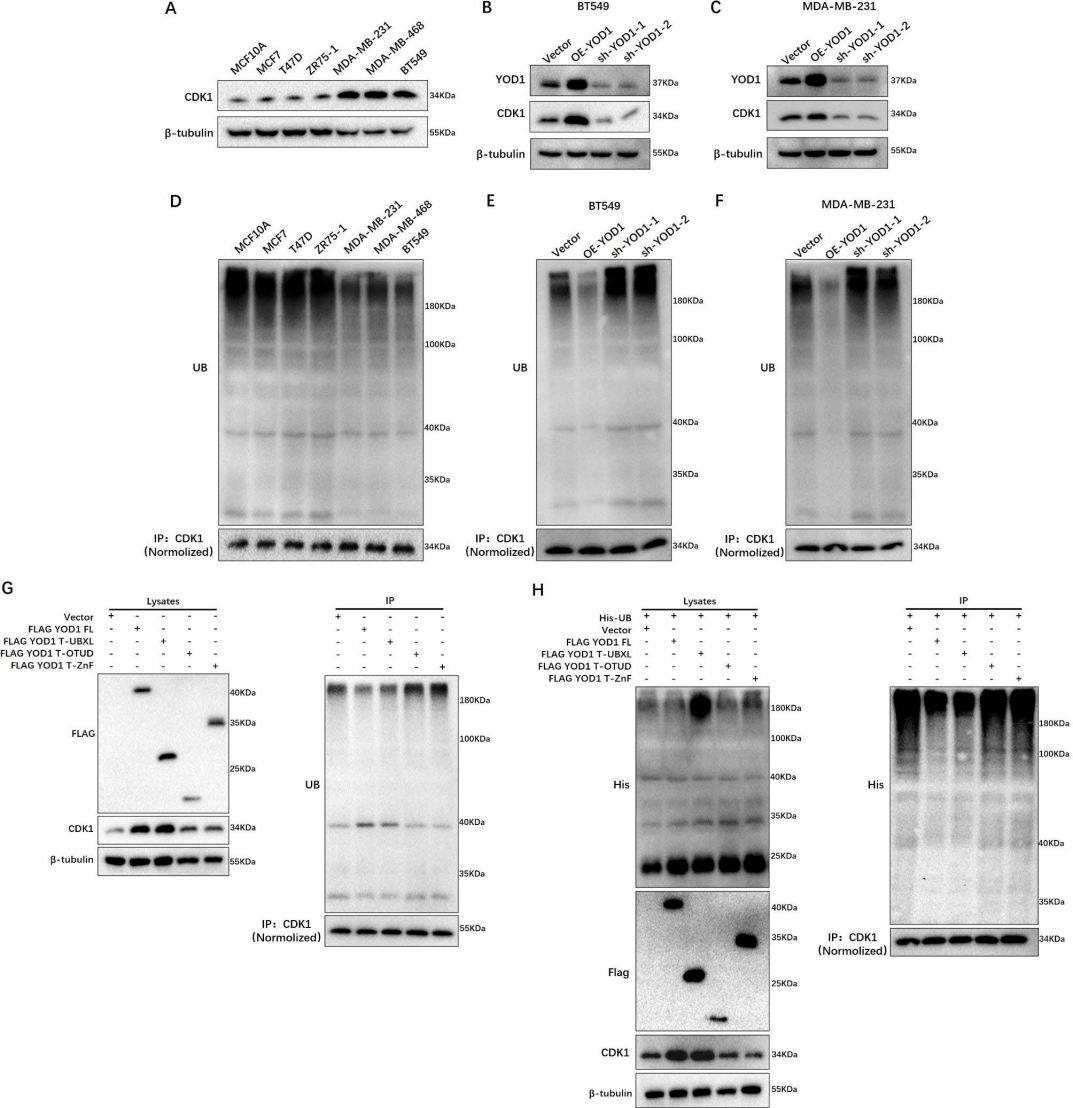
YOD1 deubiquitylates CDK1 and maintains CDK1 stability. **A**, Western blotting showed the expression of CDK1 in NTNBC cells (MCF10A, MCF7, T47D, ZR75-1) and TNBC cells (MDA-MB-231, MDA-MB-468, BT-549). **B-C**, Evaluate the expression of CDK1 when YOD1 gain and loss of function were performed in MDA-MB-231 and BT-549 cells by western blotting. **D**, Immunoprecipitation combined with western blotting showed the CDK1 ubiquitination levels in NTNBC and TNBC cells. **E-F**, Evaluate CDK1 ubiquitination levels when YOD1 gain and loss of function were performed in the indicated cells. **G**, Flag-tagged YOD1 and its truncated constructs were transfected respectively into 293T cells to evaluate expression of CDK1. The cell lysates were subjected to immunoprecipitation with anti-CDK1 antibodies to detect CDK1 ubiquitination levels. **H**, His-tagged UB and Flag-YOD1 constructs were co-transfected into 293T cells to evaluate expression of CDK1 and exogenous constructs expression. The cell lysates were subjected to immunoprecipitation with anti-CDK1 and detected exogenous ubiquitination levels

To determine the possible regulatory effect of YOD1 on CDK1, the protein expression level of endogenous CDK1 was detected by Western blotting in three TNBC cell lines including MDA-MB-231, MDA-MB-468 and BT549 that characterized YOD1 overexpression, and four NTNBC cell lines (MCF10A, MCF7, T47D, ZR75-1) as a control group. We found that CDK1 protein expression was elevated in TNBC cell lines, compared with the NTNBC cell lines (Fig. 6A). To verify this phenomenon, we tested the YOD1 and CDK1 expression in NTNBC or TNBC tissue by Immunofluorescence (Fig. S7). And then YOD1 was overexpressed or knocked down in MDA-MB-231 and BT549 to detect CDK1 protein expression. It was found that YOD1 increased CDK1 protein level, whereas YOD1 knockdown markedly decreased the CDK1 protein level (Fig. 6B C). Furthermore, YOD1 overexpression or knockdown does not affect the mRNA transcription level of CDK1, suggesting that YOD1 does not affect the stability of basal genome of CDK1, which may be a post transcriptional regulation. (Fig. S8).

Knowing that YOD1 binds to and positively regulates CDK1 protein expression, we firstly examined the level of CDK1 ubiquitination in various TNBC and NTNBC cell lines by immunoprecipitation. We observed strong ubiquitylation of CDK1 only in NTNBC cells, whereas CDK1 ubiquitylation level in TNBC cells was reduced (Fig. 6D). Furthermore, the ubiquitination level of CDK1 significantly decreased with YOD1 overexpression, whereas the ubiquitination level of CKD1 increased with YOD1 knockdown both in MDA-MB-231 and BT549 cells (Fig. 6E F). Besides, various truncated YOD1 mutants were transfected into HEK293T cells to detect the level of CDK1 ubiquitination. It was found that the level of CDK1 ubiquitination was significantly decreased compared with YOD1-T-OTUD and YOD1-T-ZnF mutant, but increased in the full-length FLAG-YOD1 and YOD1-T-UBXL mutant (Fig. 6G). These findings indicate that CDK1 bound to the Znf domain of YOD1 and then YOD1-T-OTUD was the Ub-specific domain of YOD1 to deubiquitinate CDK1 while the N-terminal domain had no deubiquitinating effect. Additionally, we co-expressed a His-tagged Ub (His-Ub) in HEK293 cells with simultaneous treatment of various YOD1 truncation mutants. Co-immunoprecipitation assay further validated that OTUD was the Ub-specific domain and Znf was the binding specificity domain of CDK1, indicating that both OTUD and Znf domains are required for YOD1 activity (Fig. 6 H).

CDK1 is involved in the control of events such as DNA replication, mRNA transcription and translation, DNA repair and cell morphogenesis, all of which are closely related to the development of breast cancer [25, 26]. Rb and 70S6K are the direct downstream proteins of CDK1 that mediate the above functions [27, 28], so that the phosphorylation of both proteins can be detected when YOD1 gains and loses the function. We found that YOD1 promoted Rb and 70S6K phosphorylation by maintaining CDK1 stability, while YOD1 knockdown reversed this effect markedly (Fig. S8.B).

Furthermore, CDK1 inhibitors (RO3360 4 μm and CDK1-IN-4 2 μm) were used and the CCK8 results showed that CDK1 inhibition completely eliminated the proliferative effect of YOD1 overexpression. Flow cytometry analysis showed that CDK1 inhibitors could completely offset the resistance to cisplatin mediated by overexpression of YOD1 (Fig. S9). These results indicated that YOD1 mainly exerts its tumor promoting effect through CDK1.

### YOD1 promotes TNBC growth and metastasis in vivo

**Fig. 7:**
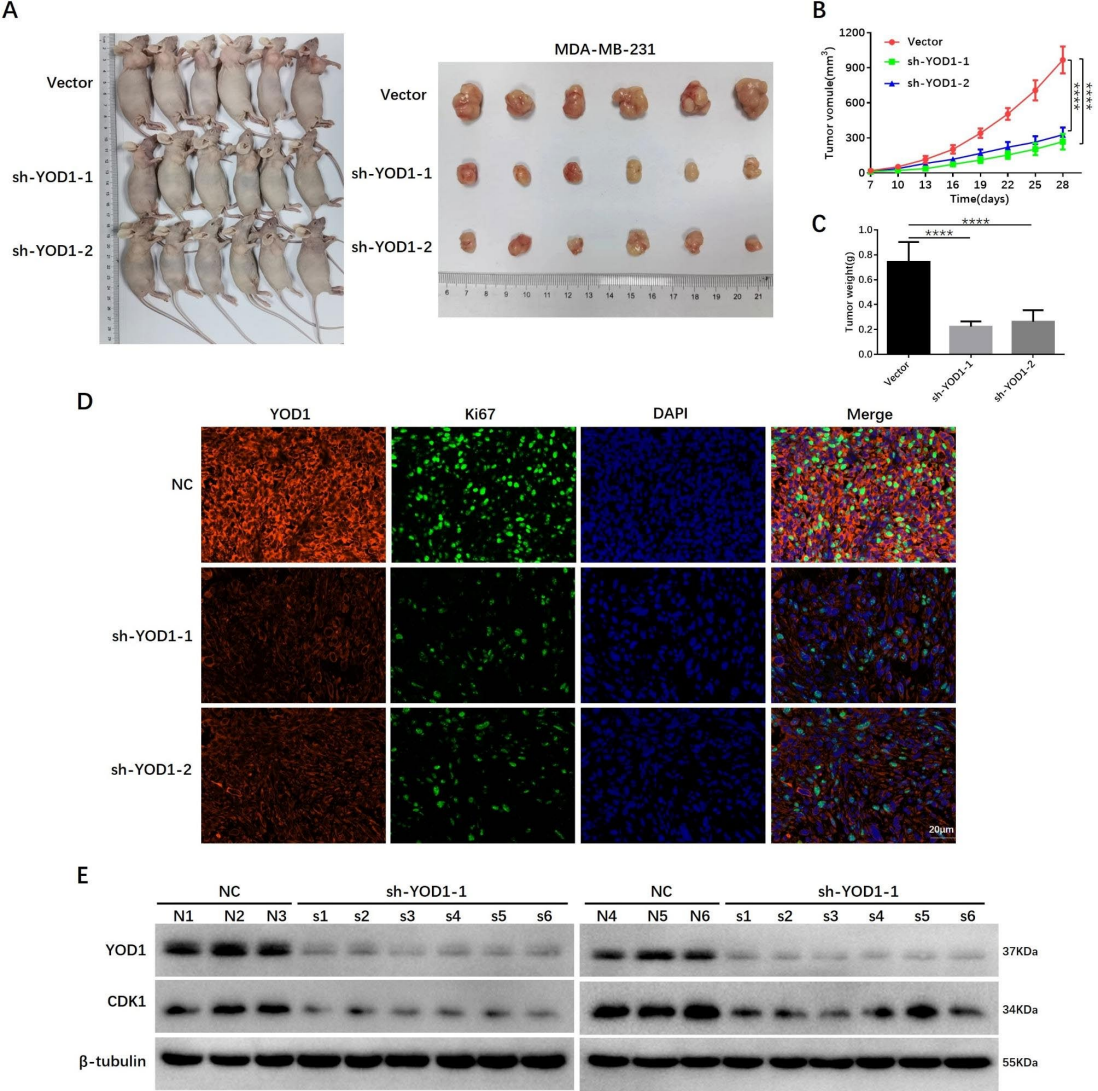
YOD1 promotes TNBC growth in vivo. **A**, 8-weeks-old female BALB/c nude mice were subjected to subcutaneous implantation into the dorsolateral side of the flank region for the indicated cell respectively. Representative images of the primary tumors from each group for twenty-eight days are shown. **B-C**, Primary tumor volume was measured every three days. Primary tumors were removed and weighed in each group. **D**, The expression of YOD1 and Ki67 in each group tumors were detected by immunohistochemistry. Scale bars, 25 μm. **E**, YOD1 and CKD1 protein expression was detected in mouse model tissue by Western blot analysis. Data are mean ± SEM. one-way ANOVA test. (****P < 0.0001)

To study the role of YOD1 in vivo, we implanted MDA-MB-231 cells with stable knockdown of YOD1 and control cells into the dorsolateral side of the flank region of BALB/c nude mice (Fig. 7A). The data were consistent with the above role of YOD1 in vitro, as manifested by the smaller tumor volume, a lower rate of tumor growth, and lighter tumor weight in YOD1 knockdown group, compared with the control group (Fig. 7B C). Cell proliferation was additionally evaluated by Ki67 positive expression and YOD1 expression of the mouse tumor tissues using triple immunofluorescence (IF). We found that the Ki67 staining intensity and cell number were decreased in YOD1 knockdown tumors (Fig. 7D). Next, we analyzed YOD1 and CDK1 expressions by Western Blotting in the tumor tissues and found that CDK1 expression was downregulated in the tumor tissues of breast tumor-bearing mice with YOD1 knockdown (Fig. 7E).

Tumor metastasis is another malignant behavior for TNBC. YOD1 knockdown MDA-MB-231 cells were injected into nude mice through the tail vein and the bioluminescent imaging showed YOD1 knockdown could significantly decrease the tumor burden and metastasis. In vitro experiments showed that the expression level of CDK1 and cell proliferation activity of TNBC gradually decreased when the concentration of ubiquitin isopeptidase inhibitor I (G5), a YOD1 inhibitor, increased [29]. Meanwhile, in vivo experiments showed that G5 could inhibit the metastasis and proliferation of TNBC cell lines (Fig. S10). To sum up, these data highlight the function and mechanism of YOD1 to facilitate breast tumor growth, while YOD1 knockdown or inhibitor could weaken TNBC growth and metastasis.

## Discussion

TNBC is the subtype with the worst prognosis of all breast cancers due to tumor heterogeneity, extreme aggressiveness and long-standing paucity of effective therapies other than the conventional therapies of surgery, radiation or chemotherapy [1, 30]. Although the innovations of omics technologies have revealed potentially actionable molecular features in some TNBC cases [30–32], there is still a lack of effective clinical therapies. Therefore, it is urgent to find more effective molecule targets specific to TNBC patients. With the continuous progress in TNBC research, accumulating evidence proves that OTUD plays a critical role in cellular processes such as deubiquitination and other biological metabolism processes of the disease [33–36]. In this study, we reported for the first time that YOD1 could be regarded as a potential prognostic biomarker or a therapeutic target for TNBC in that a higher expression of YOD1 was associated with a higher grade, a higher stage and poorer OS in patients with TNBC. Additionally, we revealed that YOD1 drove tumorigenesis by directly interacting with CDK1 and regulating its stability, suggesting that YOD1 may prove to be a potential therapeutic target for TNBC.

OTUDs are a subtype of DUBs, which control the function and stability of proteins by removing Ub chains from a target protein in a wide range of intracellular processes, including endoplasmic reticulum -associated degradation pathway, cell cycle progression and signal transduction [12, 37–39]. Numerous studies have demonstrated that the OTU subfamily played a key role in tumor growth, invasion and metastasis in different cancer models through its participation in multiple steps of intracellular processes, especially for TNBC [17, 40]. OTUD7B interacts with, deubiquitylate, stabilizes estrogen receptor α in a deubiquitylation activity-dependent manner, and promotes breast cancer cell proliferation, resulting in poor prognosis [17]. Phosphorylation of OTULIN (OTU deubiquitinase with linear linkage specificity) upon DNA damage drives β-catenin activation, thus promoting drug resistance in TNBC [41]. Similarly, based on the analysis on TCGA, we observed a correlation between the expression of OTUD family members and the prognosis of TNBC patients. Differently, we found that YOD1 showed a strong correlation with poor prognosis in TNBC patients. Previous studies have reported the regulatory role of YOD1 in cancer developments, such as, YOD1 suppresses the malignant development of ovarian cancer via binding to miR-4429 [42] and YOD1 serves as a potential prognostic biomarker for pancreatic cancer through cell adhesion molecules, p53, Hippo, TGF-β and other pathways [43]. However, no study has reported the effect and mechanism of YOD1 on the development of TNBC. It was found in our study that YOD1 upregulation was negatively correlated with the clinical stage of TNBC. Besides, elevated expression of YOD1 was associated with TNBC cell proliferation, invasiveness, drug resistance and cell cycle disorder. All these results suggest that YOD1 plays a critical role in the occurrence and progression of NTBC.

YOD1 is a member of the deubiquitinating enzyme family, whose protein comprises three conserved domains: N-terminal UBX domain, central otubain domain and C-terminal C2H2-type Znf domain [24, 33]. Some studies have reported that protein domains, accessories to the catalytic core, are required for YOD1 function [19, 33, 43]. YOD1 is associated with p97 to facilitate protein dislocation, in which the dominant negative effect is dependent on the UBX and Zinc finger domains, appended to the N- and C-terminus of the catalytic otubain core domain, respectively [24]. The N-terminal UBX domain of YOD1 is essential to mediate Ub ligase TRAF6 interaction to counteract IL-1 signaling to NF-κB pathway [19]. Our mechanistic study demonstrated that YOD1-mediated promotion with deubiquitinating was dependent on its Znf domain, which acts by directly binding with CDK1, and scavenge Ub by OTUD domain with canonical enzymatic activity regulation of cell cycle, indicating that YOD1 maintains CDK1 stability and de-polyubiquitylates CDK1 to regulate the biological process for the initiation and progression of TNBC, which may be the potential function and exact mechanism of YOD1 in TNBC.

CDK1 belongs to the cyclin-dependent protein kinase family, which are involved in numerous important processes in cell biology, including cell proliferation, apoptosis and cell cycle [25, 26, 44]. Numerous studies have demonstrated that CKD1, as a key cell cycle regulator to control G2/M, is involved in the development of TNBC [45–47]. Previous investigators have found that cyclin CDK1, CCNB1 and CCNA2 are commonly overexpressed in TNBC and correlated with worse OS in breast cancer, so they could act as novel biomarkers for TNBC treatment [48]. Combined BCL-XL and CDK1/2/4 inhibition induces cell-cycle arrest, apoptosis and a shorter OS rate of TNBC patients [49]. It was reported that inhibition of CDK1 with nanoparticle carrier (NPsiCDK1) decreased the viability and apoptosis of c-Myc overexpressed TNBC cells [50]. Consistent with the above-mentioned expression of CDK1, our study also identified that CDK1 overexpression was associated with YOD1 both in vitro and in vivo, playing a regulatory role in cell cycle, proliferation, migration and invasion of tumor cells, and drug resistance. It would be of interest to test the combined effect of YOD1 and CDK1 in TNBC models. Importantly, we are the first to have shown that YOD1 expression could induce CDK1 de-polyubiquitylation, thus revealing the potential underlying oncogenic role of YOD1.

Rb, one of the important elements of E2F complex, is essential for coordinating the expression of CDK1 and CDK1-related cyclins. Furthermore, CDK1-related cyclins are usually unstable and their regulation is mainly through ubiquitination [27, 28]. P70S6K, the main mTOR substrate, is involved in diverse cellular functions, also dependent on CDK1 during mitosis [28]. It was found in our study that the phosphorylation of Rb and 70S6K could be detected when YOD1 gained and lost the function (Fig. S6), indicating the possible downstream signaling pathway. In addition, CDK1 inhibitors could eliminate the effect of YOD1 overexpression (the proliferation and resistance to cisplatin), which further elucidates the tumor promoting mechanism of the YOD1-CDK1 axis.

## Conclusion

Our study comprehensively assessed the role of YOD1 in TNBC progression and metastasis by analysis of patients’ tissue samples, in vitro and in vivo animal experiments. High expression of YOD1 was a critical feature of TNBC and was associated with poor prognosis of TNBC. YOD1 promoted TNBC progression in a manner dependent on its catalytic activity through binding with CDK1, leading to de-polyubiquitylation of CDK1. CDK1 stabilization could further promote tumor deterioration and poor prognosis. Interference with YOD1 expression or YOD1 inhibitor could suppress the above-mentioned deterioration with tumor progression. For the first time, we defined the regulatory functions of the OTUD family of YOD1 proteins in TNBC. More importantly, our study provides evidence-based clues for the use of YOD1 to develop novel diagnostics and therapeutic targets for TNBC.

### Electronic supplementary material

Below is the link to the electronic supplementary material.

Supplementary Material 1: Figure S1. Correlation analysis of each OTUD family members with prognoses of breast cancer based on TCGA database. A, Correlation analysis between OTUD family members expression and 10-year survival of breast cancer based on publicly available TCGA database. B, YOD1 protein levels in 2 TNBC tissues and 9 none-TNBC tissues by Western blotting

Supplementary Material 2: Figure S2. Expression of YOD1 was investigated in the NTNBC and TNBC cell lines. A, YOD1 mRNA levels were quantified in the indicated cells by qRT-PCR. B, YOD1 protein levels were detected in the indicated cells by Western blotting. C, YOD1 expression were evaluated in the indicated cells using immunohistochemistry. Data are mean ± SEM. Scale bars, 20 μm

Supplementary Material 3: Figure S3. The detection of apoptosis level in MCF-10 A by flow cytometry with YOD1 overexpression or knockdown. A, Validated the protein expression of YOD1 gain and loss in MCF-10 A cells by western blotting assays. B, Flow cytometry assays were performed to evaluate the effects of YOD1 gain and loss of function on the 10 µM cisplatin or 50 nM paclitaxel induced apoptosis of the indicated MCF-10 A cells. Data are mean ± SEM. one-way ANOVA test. (*P < 0.05, **P < 0.01, ns: no significance)

Supplementary Material 4: Figure S4. The immunoprecipitates of pcDNA3.1-YOD1-FLAG group or pcDNA3.1- group were analyzed by LC-MS/MS

Supplementary Material 5: Figure S5. YOD1 interacts with C-terminal Znf domain of CDK1 in 293T cells. A, VN173-YOD1 and VC155-CDK1 were co-transfected into 293T cells and detected by BiFC. The signal was detected when any other combination of VN173-YOD1 and VC155-CDK1 constructs was used. B, Different YOD1 truncated constructs of YOD1 (T-UBXL, T-OTUD, T-ZnF) and VC155-CDK1 constructs were co-transfected into 293T cells and detected the BiFC signal. Scale bars, 50 μm

Supplementary Material 6: Figure S6. Detection of the interaction between YOD1 and CKD1 by GST pulldown. A, Results of Coomassie blue staining; B, Results of WB

Supplementary Material 7: Figure S7. YOD1 over-expression is associated with CDK1 over-expression in human breast cancer specimens. Detected the YOD1 and CDK1 expression by immunohistochemistry. Scale bars, 100 μm or 20 μm

Supplementary Material 8: Figure S8. YOD1 promoted Rb and 70S6K phosphorylation by maintaining CDK1 stability. A, No change in the mRNA level of CDK1 was found When YOD1 is overexpressed or knocked down. B, The expression changes of Rb and 70S6K phosphorylation in YOD1 upregulated or downregulated cells were detected. The protein levels and their phosphorylation levels were detected by Western blotting

Supplementary Material 9: Figure S9. Inhibition of CDK1 activity offsets the tumor promoting effect of YOD1. A, The use of CDK1 inhibitors can significantly inhibit the proliferation promoting effect of YOD1. B, Flow cytometry analysis of apoptosis suggests that CDK1 inhibitors could significantly offset the resistance to cisplatin. Statistical analysis was performed using one-way ANOVA test. Mean ± SEM (**P < 0.01, ***P < 0.001, and****P < 0.0001)

Supplementary Material 10: Figure S10. YOD1 inhibitor (G5) or knockdown inhibits CDK1 activity and TNBG cell line proliferation and metastasis ability. A, The expression level of CDK1 protein changes when the concentration of G5 (0, 10, 50, 100, 200nM) changes. B, The activity of TNBC cells changes when the concentration of G5 changes. C, Knocking down YOD1 or using YOD1 inhibitor (G5) can inhibit the metastasis and proliferation of TNBC cell lines. Statistical analysis was performed using one-way ANOVA test. Mean ± SEM (* p < 0.05, *** p < 0.001, and****P < 0.0001)

Supplementary Material 11: Figure S11. Original, uncropped images of blot results(Fig. 1C and SFig 1B)

Supplementary Material 12: Figure S12. Original, uncropped images of blot results(SFig 2B, Fig. 2B and C, SFig 3 A, Fig. 4B-C, D-E and G, and Fig. 5B-D)

Supplementary Material 13: Figure S13. Original, uncropped images of blot results(Figs. 6A-H and 7E)

Supplementary Material 14: Figure S14. Original, uncropped images of blot results(SFig 8B, SFig 10 A)

Supplementary Material 15: Supplementary Table 1 and Supplementary Table 2

Supplementary Material 16: Peptides of LC-MS

Supplementary Material 17: Proteins of LC-MS

## Data Availability

The authors declare that all data and materials supporting the findings of this study are available in this article and its supplementary files.

## Abbreviations

YOD1: OTU Domain-Containing Protein 2
TNBC: Triple-negative breast cancer
NTNBC: Non-triple-negative breast cancer
ER: Estrogen receptor
PR: Progesterone receptor
HER2: Human epidermal growth factor receptor 2
DUBs: Deubiquitinating enzymes
OTU: Ovarian-tumor related protease
OTUDs: OTU domain-containing proteins
TGF-β: Transforming growth factor-bet
CDK1: Cyclin Dependent Kinase 1
PTEN: Phosphatase and tensin homolog
qRT-PCR: Quantitative real time polymerase chain reaction
HRP: Horseradish Peroxidase
IHC: Immunohistochemical
DAPI: 4’,6-diamidino-2-phenylindole
LC-MS: Liquid chromatography-mass spectrometry
TUNEL: Terminal dexynucleotidyl transferase-mediated dUTP nick end labeling
PI: Propidium iodide
p53: Tumor protein p53
P97: Valosin containing protein
NF-κB: Nuclear Factor NF-Kappa-B
BCLXL: BCL2 Like 1
E2F: E2F Transcription Factor
P70S6K: Ribosomal Protein S6 Kinase
TRAF6: TNF Receptor Associated Factor 6
