# Supplementary figures and images for: Deubiquitylase YOD1 regulates CDK1 stability and drives triple-negative breast cancer tumorigenesis
Source: J Exp Clin Cancer Res. 2023 Sep 4;42:228. doi: 10.1186/s13046-023-02781-3 (PMC10478497; doi:10.1186/s13046-023-02781-3)

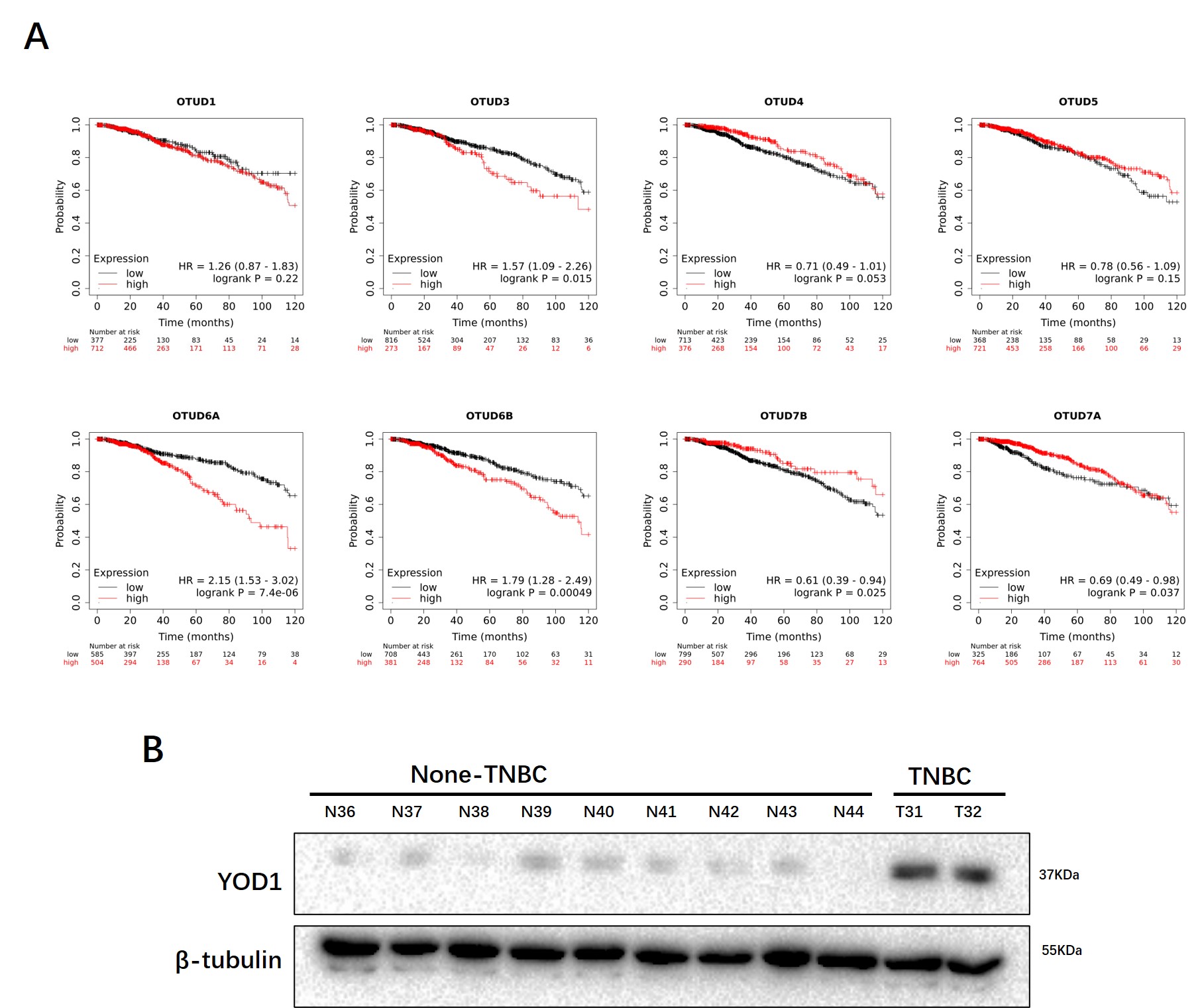

Supplement: Supplementary file 1 — Supplementary Material 1: Figure S1. Correlation analysis of each OTUD family members with prognoses of breast cancer based on TCGA database. A, Correlation analysis between OTUD family members expression and 10-year survival of breast cancer based on publicly available TCGA database. B, YOD1 protein levels in 2 TNBC tissues and 9 none-TNBC tissues by Western blotting [file 13046_2023_2781_MOESM1_ESM.jpg]

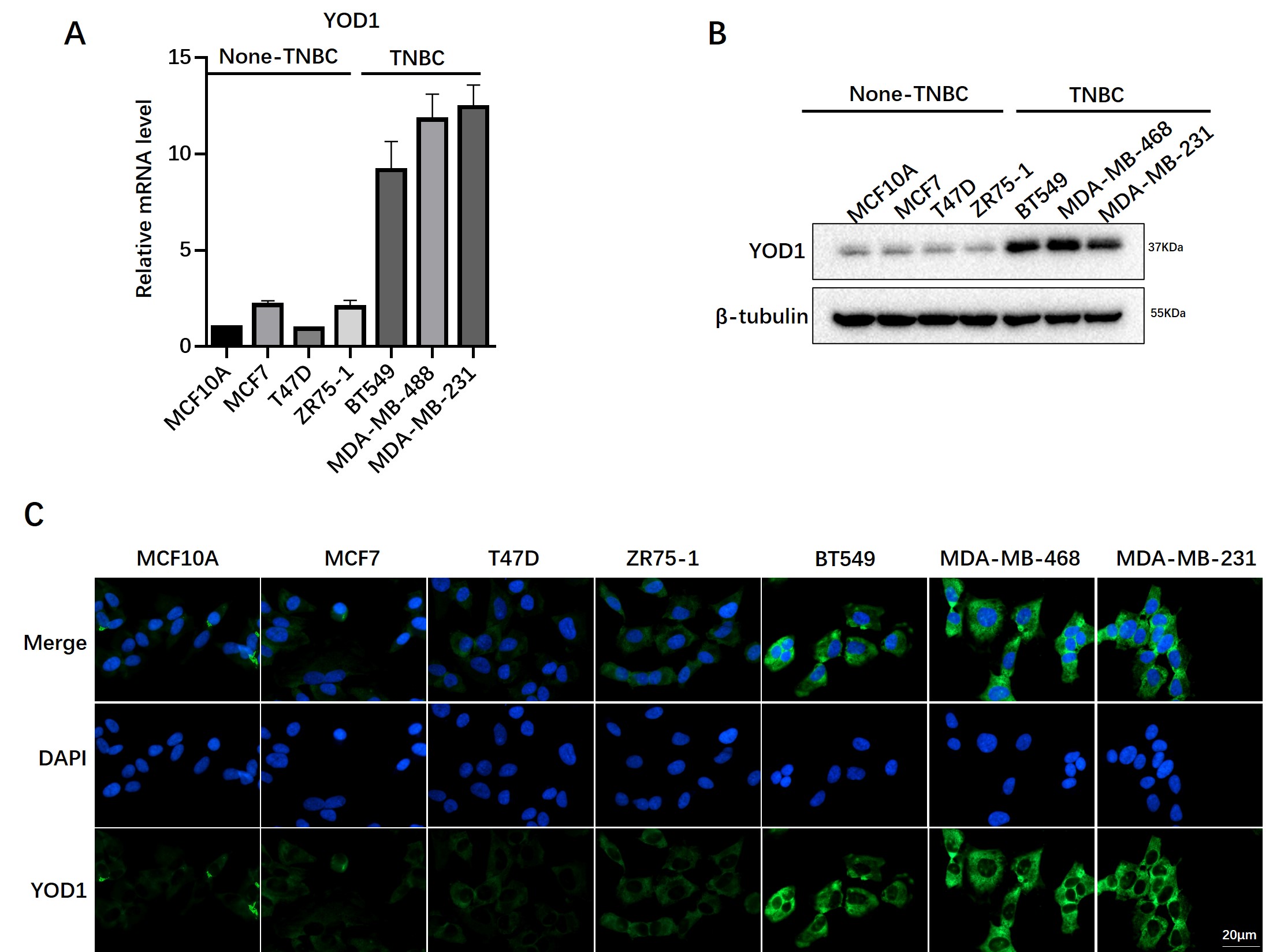

Supplement: Supplementary file 2 — Supplementary Material 2: Figure S2. Expression of YOD1 was investigated in the NTNBC and TNBC cell lines. A, YOD1 mRNA levels were quantified in the indicated cells by qRT-PCR. B, YOD1 protein levels were detected in the indicated cells by Western blotting. C, YOD1 expression were evaluated in the indicated cells using immunohistochemistry. Data are mean ± SEM. Scale bars, 20 μm [file 13046_2023_2781_MOESM2_ESM.jpg]

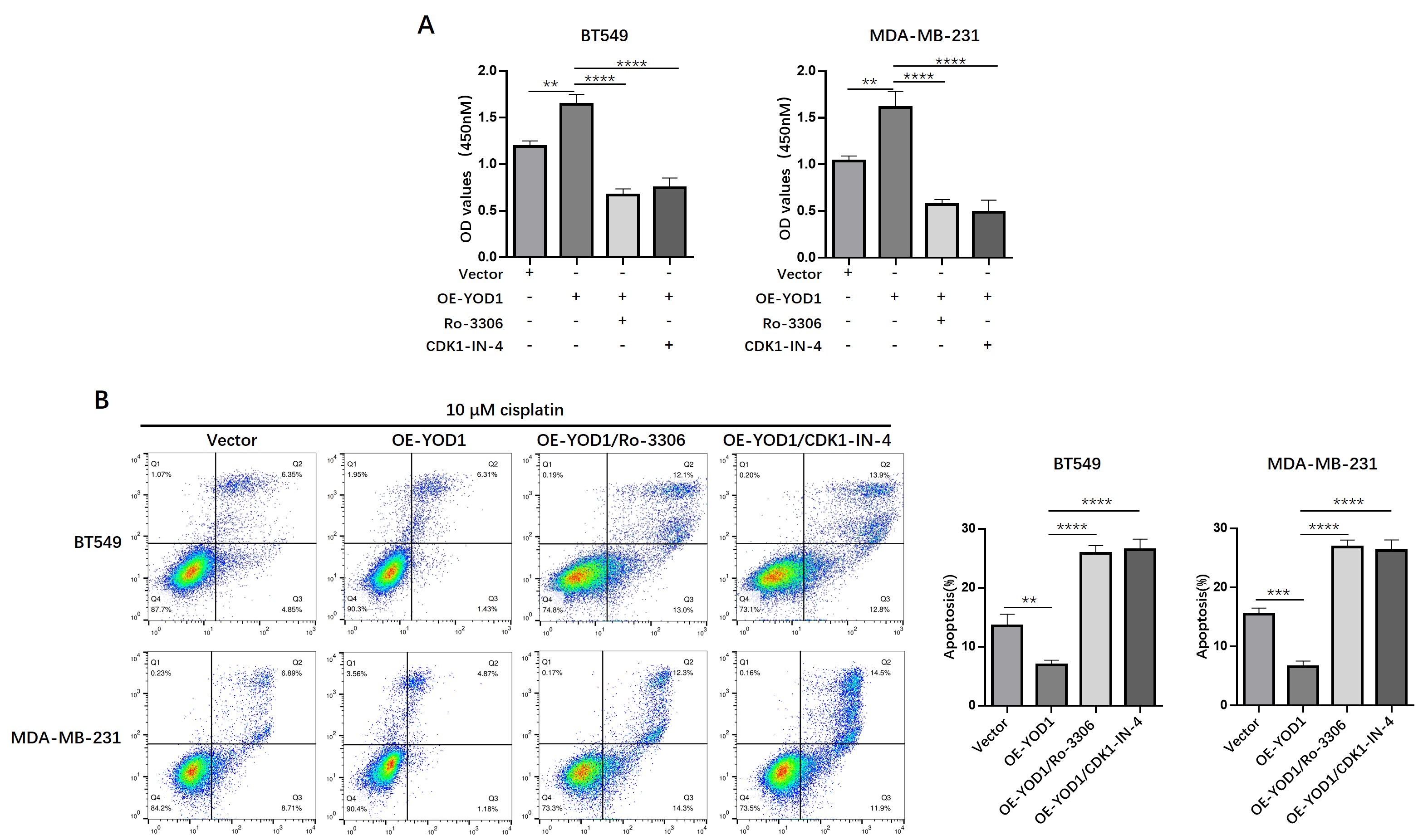

Supplement: Supplementary file 3 — Supplementary Material 3: Figure S3. The detection of apoptosis level in MCF-10 A by flow cytometry with YOD1 overexpression or knockdown. A, Validated the protein expression of YOD1 gain and loss in MCF-10 A cells by western blotting assays. B, Flow cytometry assays were performed to evaluate the effects of YOD1 gain and loss of function on the 10 µM cisplatin or 50 nM paclitaxel induced apoptosis of the indicated MCF-10 A cells. Data are mean ± SEM. one-way ANOVA test. (*P < 0.05, **P < 0.01, ns: no significance) [file 13046_2023_2781_MOESM3_ESM.jpg]

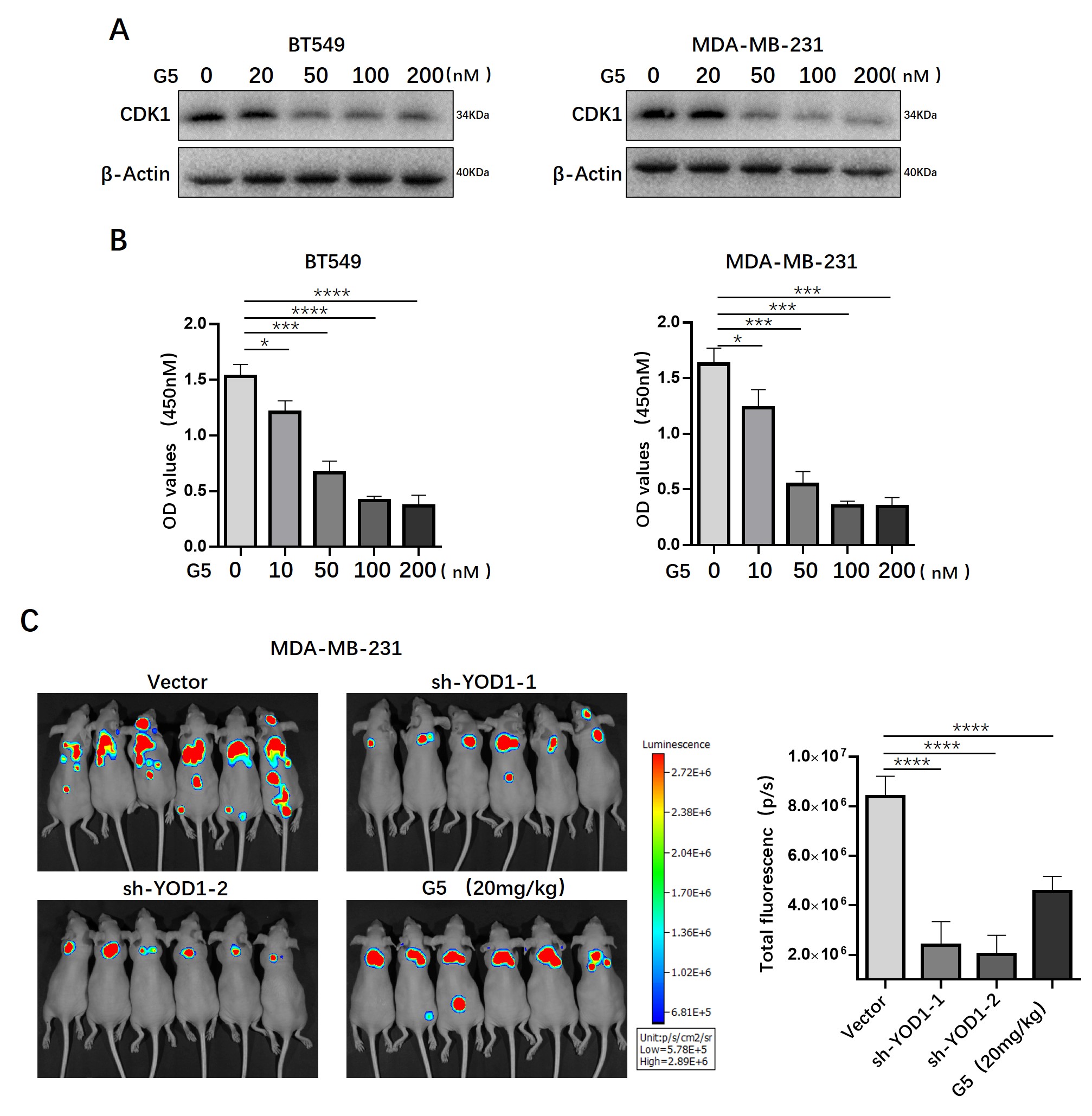

Supplement: Supplementary file 4 — Supplementary Material 4: Figure S4. The immunoprecipitates of pcDNA3.1-YOD1-FLAG group or pcDNA3.1- group were analyzed by LC-MS/MS [file 13046_2023_2781_MOESM4_ESM.jpg]

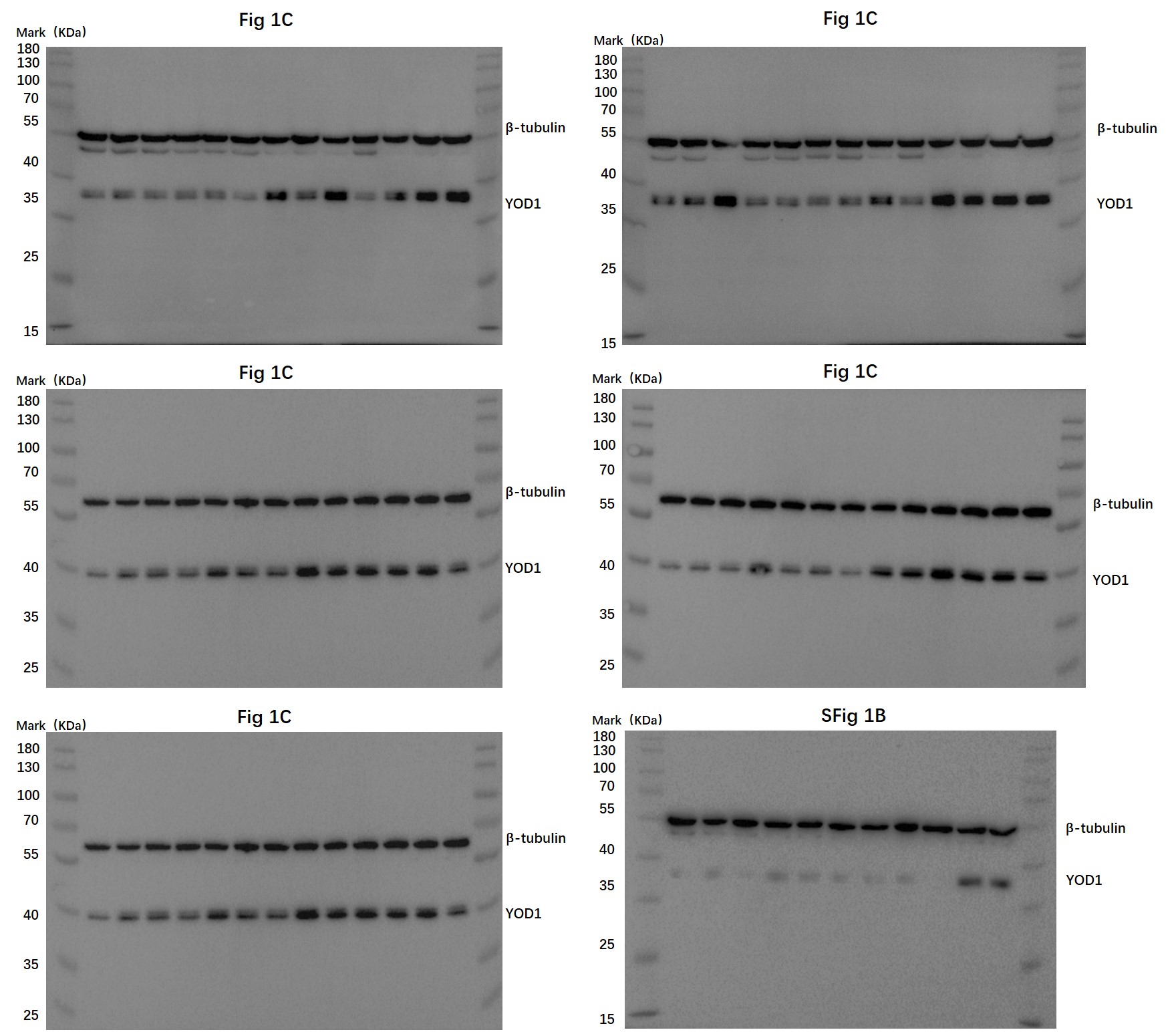

Supplement: Supplementary file 5 — Supplementary Material 5: Figure S5. YOD1 interacts with C-terminal Znf domain of CDK1 in 293T cells. A, VN173-YOD1 and VC155-CDK1 were co-transfected into 293T cells and detected by BiFC. The signal was detected when any other combination of VN173-YOD1 and VC155-CDK1 constructs was used. B, Different YOD1 truncated constructs of YOD1 (T-UBXL, T-OTUD, T-ZnF) and VC155-CDK1 constructs were co-transfected into 293T cells and detected the BiFC signal. Scale bars, 50 μm [file 13046_2023_2781_MOESM5_ESM.jpg]

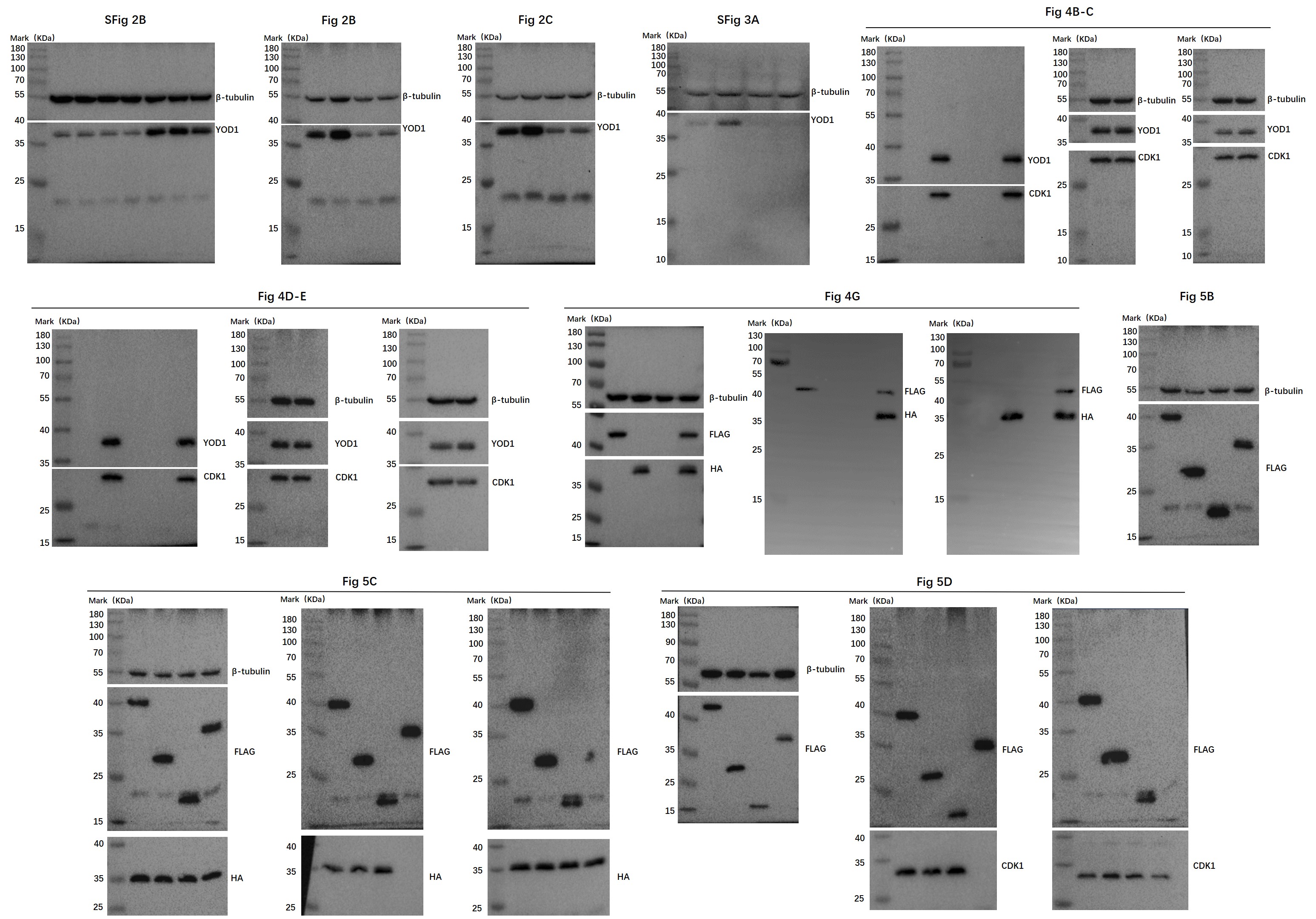

Supplement: Supplementary file 6 — Supplementary Material 6: Figure S6. Detection of the interaction between YOD1 and CKD1 by GST pulldown. A, Results of Coomassie blue staining; B, Results of WB [file 13046_2023_2781_MOESM6_ESM.jpg]

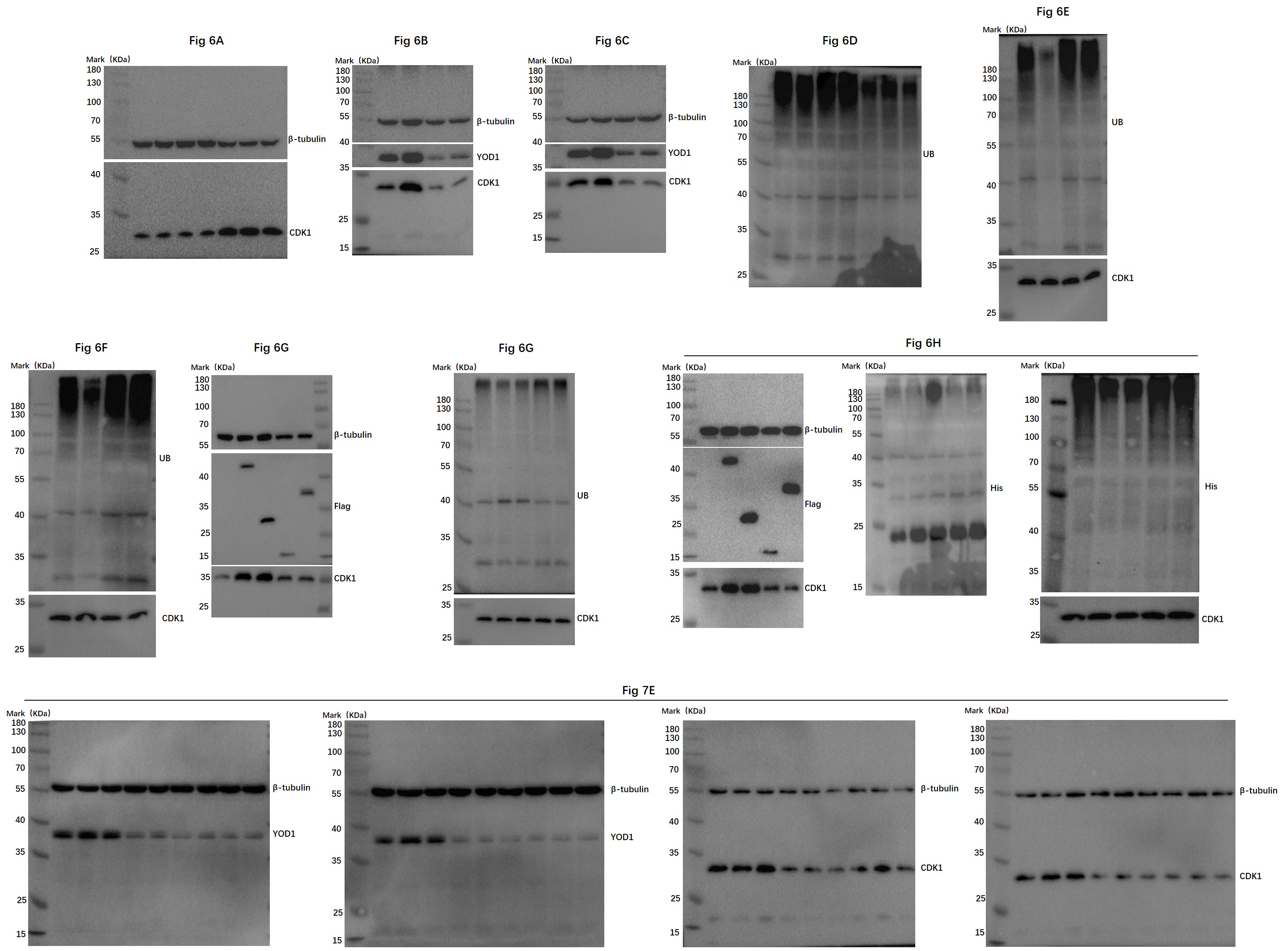

Supplement: Supplementary file 7 — Supplementary Material 7: Figure S7. YOD1 over-expression is associated with CDK1 over-expression in human breast cancer specimens. Detected the YOD1 and CDK1 expression by immunohistochemistry. Scale bars, 100 μm or 20 μm [file 13046_2023_2781_MOESM7_ESM.jpg]

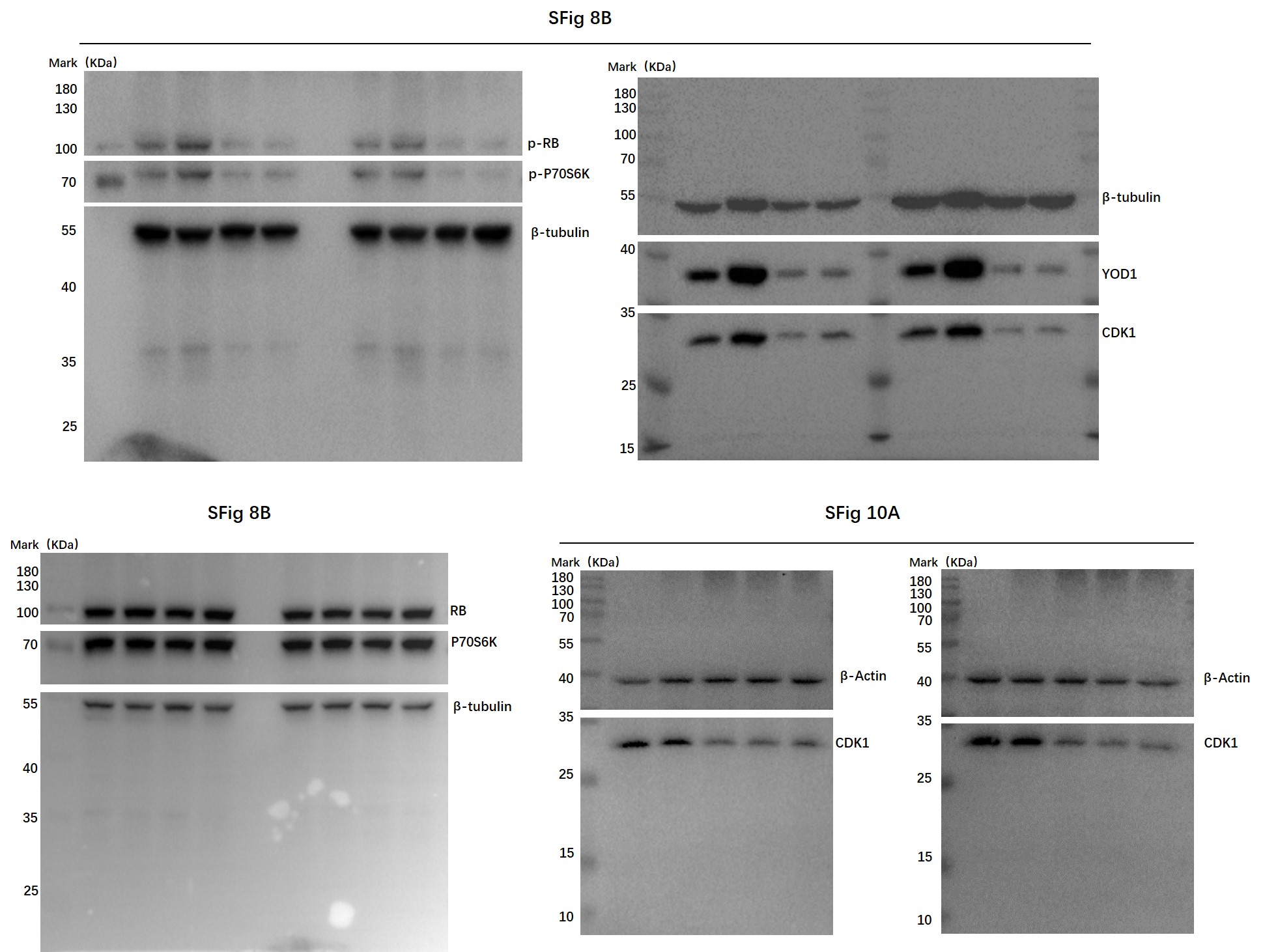

Supplement: Supplementary file 8 — Supplementary Material 8: Figure S8. YOD1 promoted Rb and 70S6K phosphorylation by maintaining CDK1 stability. A, No change in the mRNA level of CDK1 was found When YOD1 is overexpressed or knocked down. B, The expression changes of Rb and 70S6K phosphorylation in YOD1 upregulated or downregulated cells were detected. The protein levels and their phosphorylation levels were detected by Western blotting [file 13046_2023_2781_MOESM8_ESM.jpg]

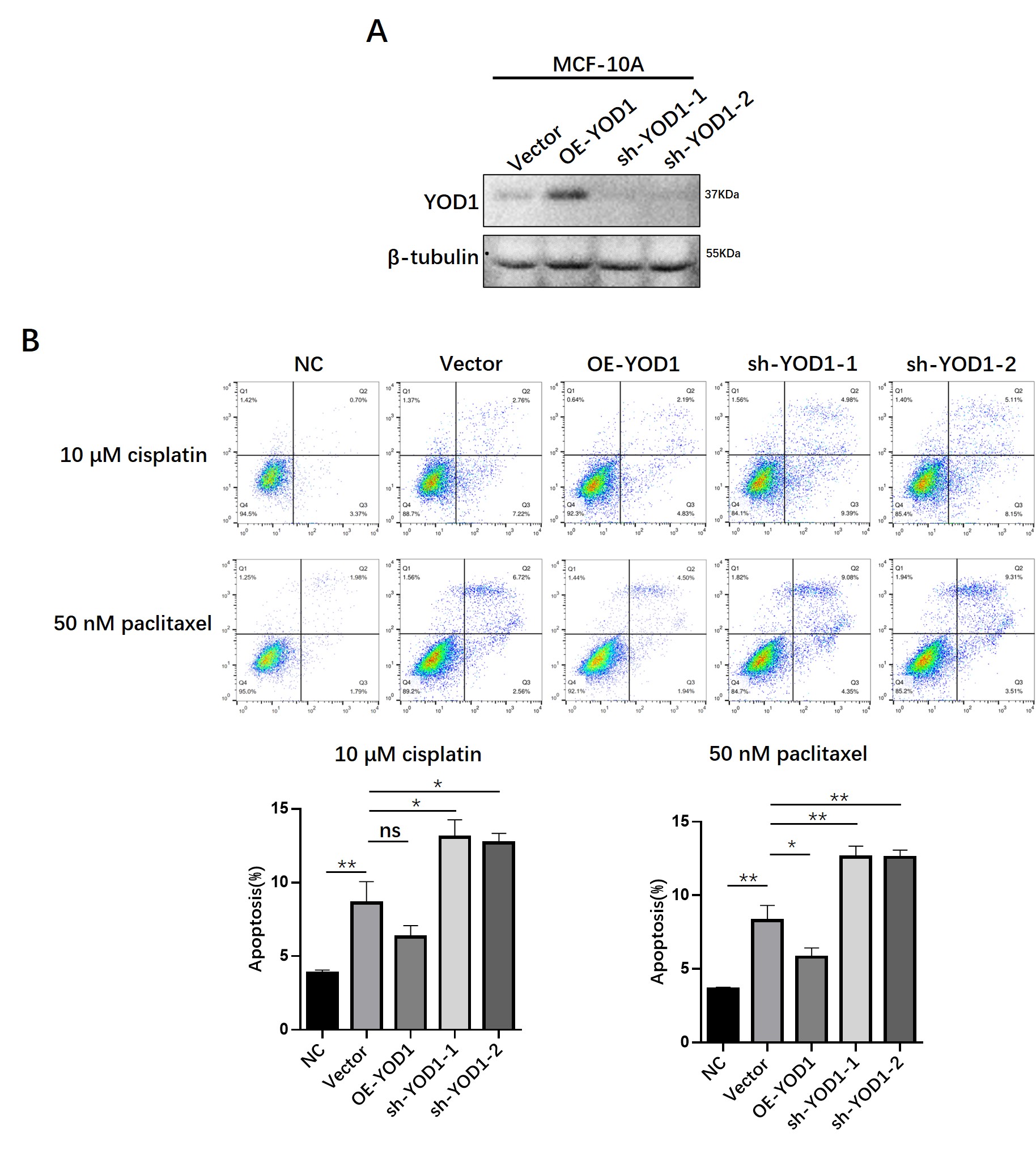

Supplement: Supplementary file 9 — Supplementary Material 9: Figure S9. Inhibition of CDK1 activity offsets the tumor promoting effect of YOD1. A, The use of CDK1 inhibitors can significantly inhibit the proliferation promoting effect of YOD1. B, Flow cytometry analysis of apoptosis suggests that CDK1 inhibitors could significantly offset the resistance to cisplatin. Statistical analysis was performed using one-way ANOVA test. Mean ± SEM (**P < 0.01, ***P < 0.001, and****P < 0.0001) [file 13046_2023_2781_MOESM9_ESM.jpg]

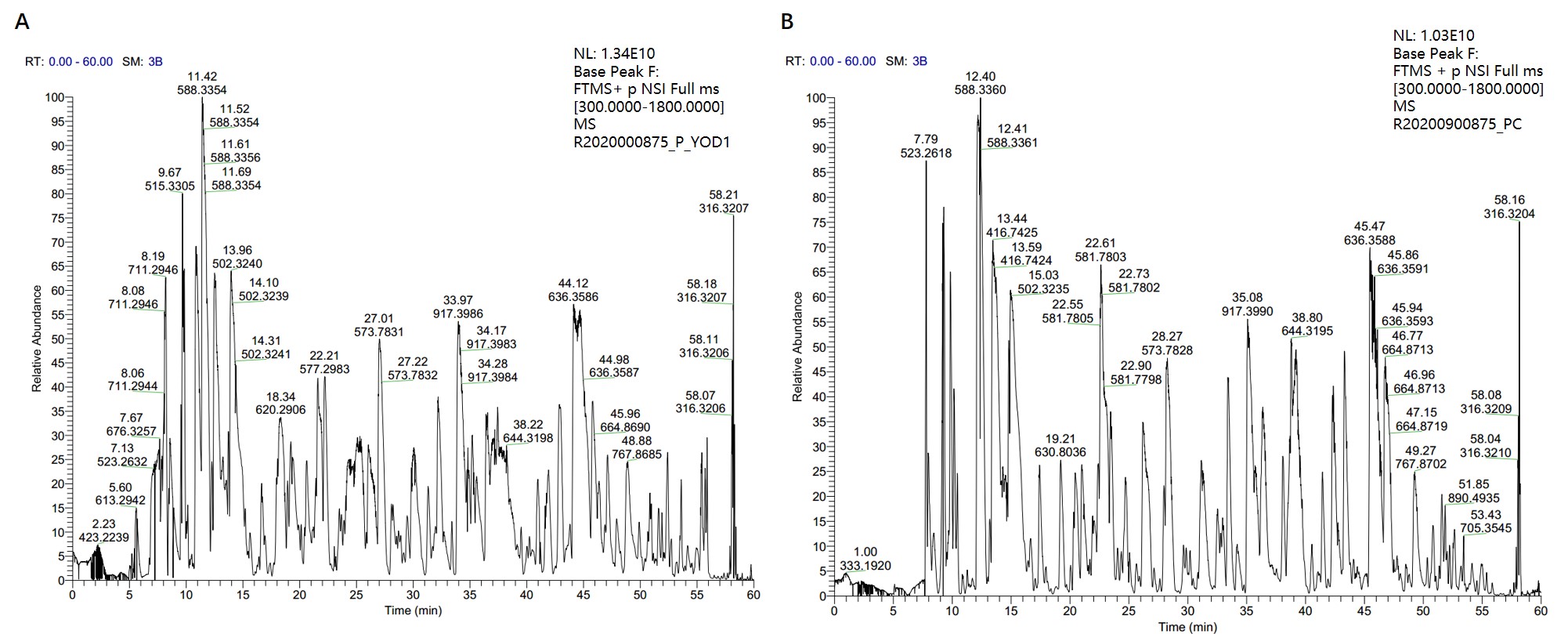

Supplement: Supplementary file 10 — Supplementary Material 10: Figure S10. YOD1 inhibitor (G5) or knockdown inhibits CDK1 activity and TNBG cell line proliferation and metastasis ability. A, The expression level of CDK1 protein changes when the concentration of G5 (0, 10, 50, 100, 200nM) changes. B, The activity of TNBC cells changes when the concentration of G5 changes. C, Knocking down YOD1 or using YOD1 inhibitor (G5) can inhibit the metastasis and proliferation of TNBC cell lines. Statistical analysis was performed using one-way ANOVA test. Mean ± SEM (* p < 0.05, *** p < 0.001, and****P < 0.0001) [file 13046_2023_2781_MOESM10_ESM.jpg]

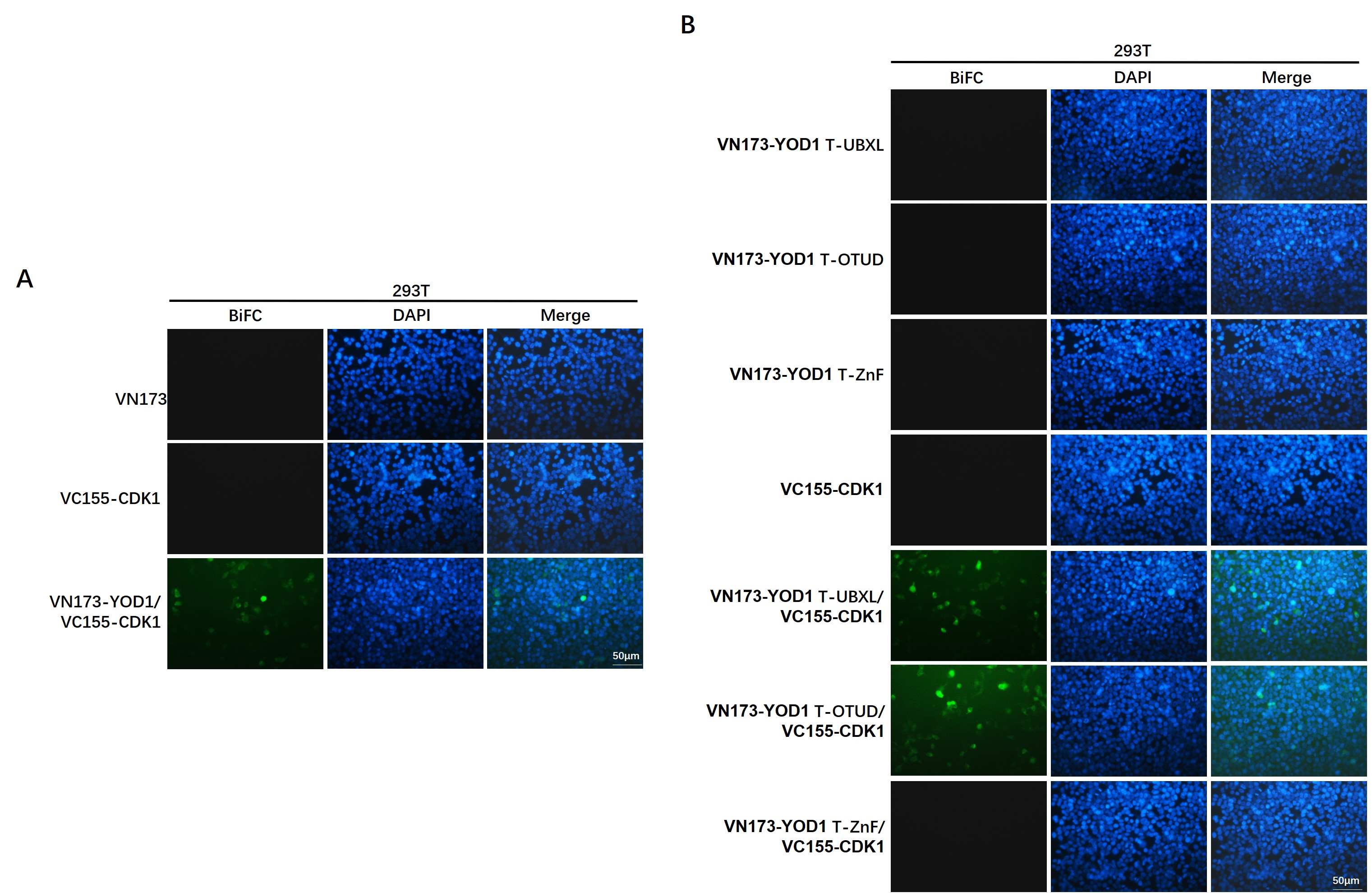

Supplement: Supplementary file 11 — Supplementary Material 11: Figure S11. Original, uncropped images of blot results(Fig. 1C and SFig 1B) [file 13046_2023_2781_MOESM11_ESM.jpg]

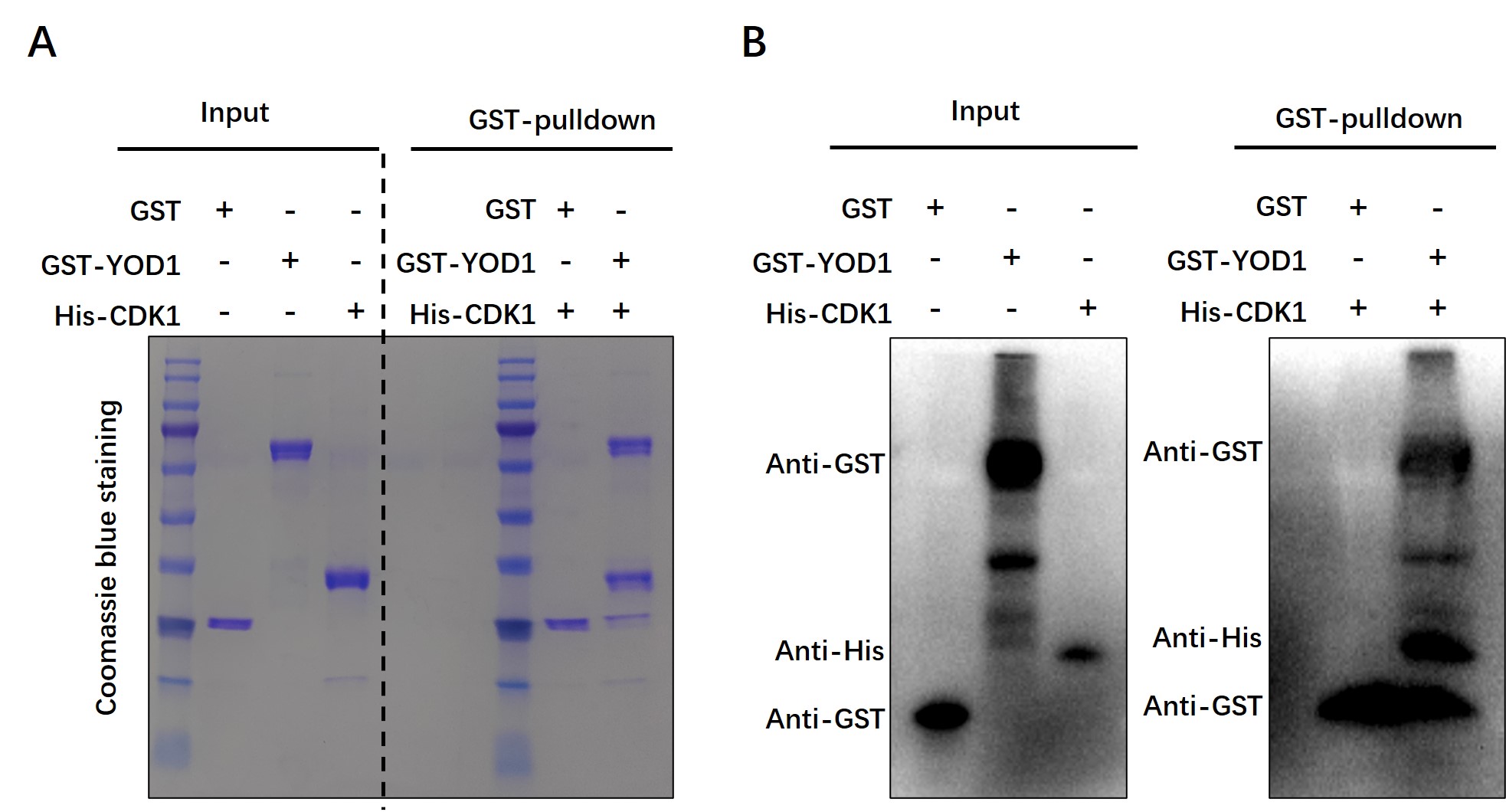

Supplement: Supplementary file 12 — Supplementary Material 12: Figure S12. Original, uncropped images of blot results(SFig 2B, Fig. 2B and C, SFig 3 A, Fig. 4B-C, D-E and G, and Fig. 5B-D) [file 13046_2023_2781_MOESM12_ESM.jpg]

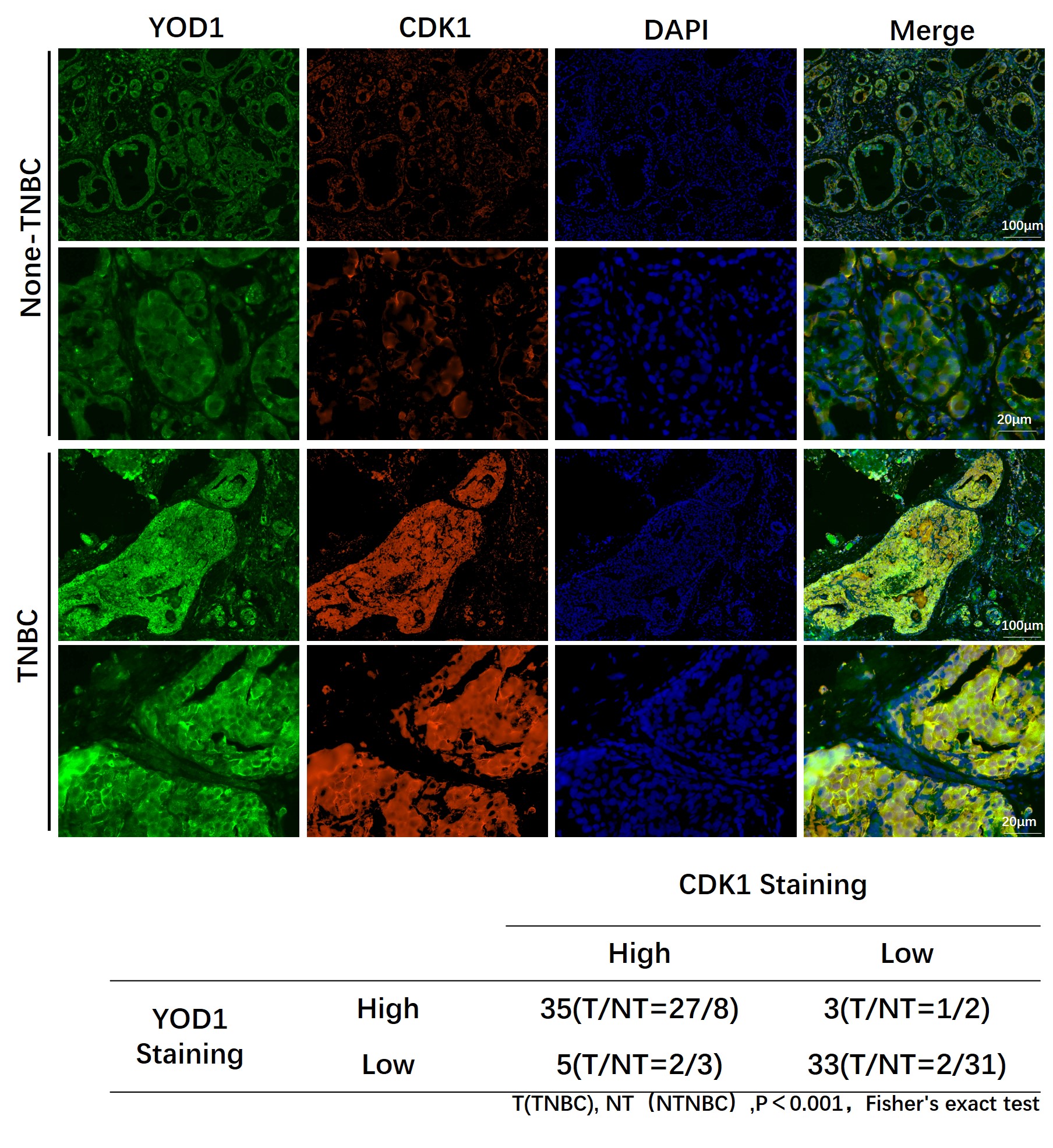

Supplement: Supplementary file 13 — Supplementary Material 13: Figure S13. Original, uncropped images of blot results(Figs. 6A-H and 7E) [file 13046_2023_2781_MOESM13_ESM.jpg]

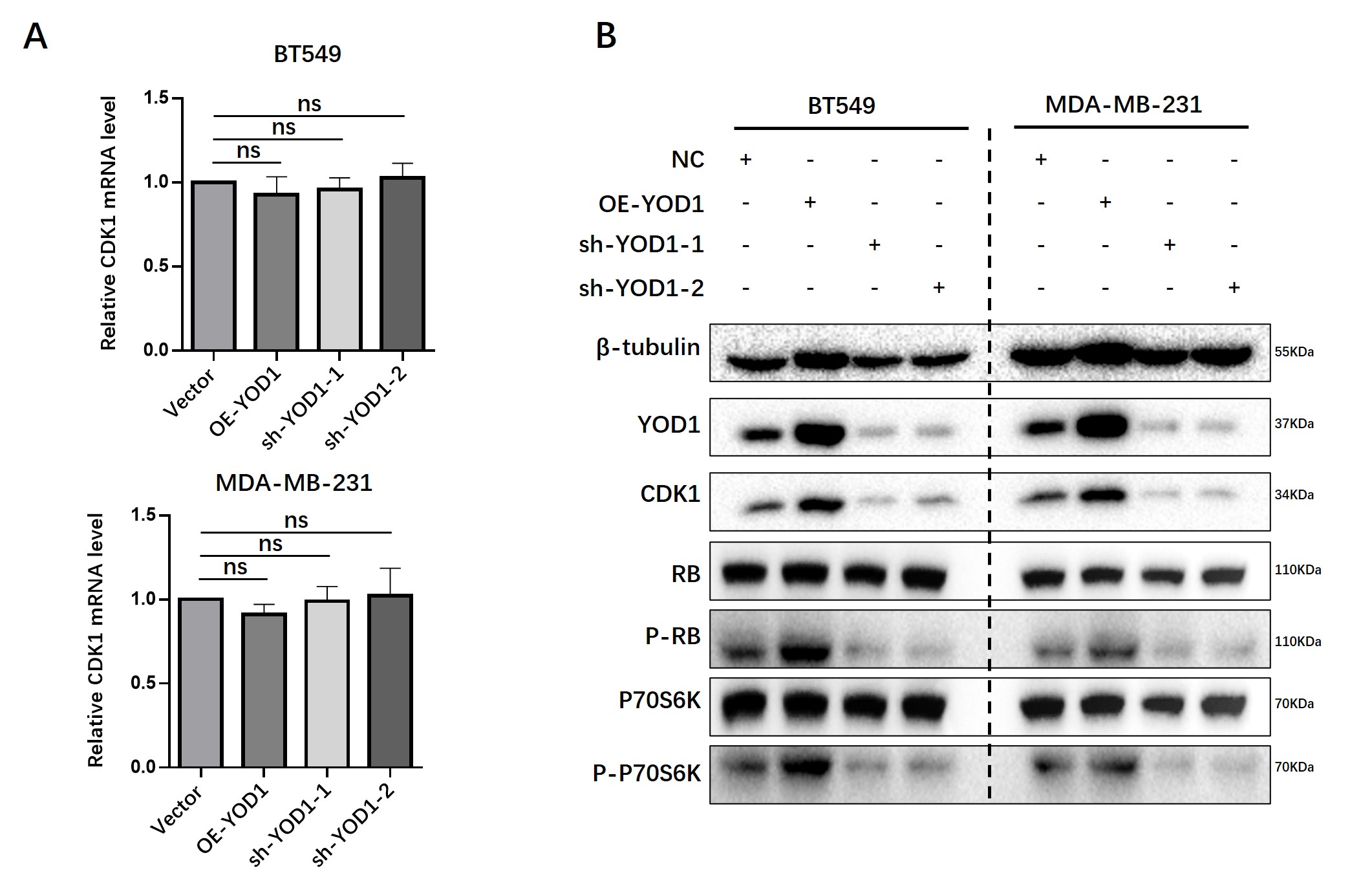

Supplement: Supplementary file 14 — Supplementary Material 14: Figure S14. Original, uncropped images of blot results(SFig 8B, SFig 10 A) [file 13046_2023_2781_MOESM14_ESM.jpg]
