# Supplementary material for: Deubiquitylase YOD1 regulates CDK1 stability and drives triple-negative breast cancer tumorigenesis
Source: J Exp Clin Cancer Res. 2023 Sep 4;42:228. doi: 10.1186/s13046-023-02781-3 (PMC10478497; doi:10.1186/s13046-023-02781-3)
Supplement: Supplementary file 15 — Supplementary Material 15: Supplementary Table 1 and Supplementary Table 2 [file 13046_2023_2781_MOESM15_ESM.docx]

| Supplementary table 1. Primer for PCR or single-stranded DNA for anneal | |
| --- | --- |
| [Name](javascript:;) | Sequence |
| sh-YOD1-1F | CCGGCAGAAAGGATTAACTGGACAATTCAAGACGTTGTCCAGTTAATCCTTTCTGTTTTTTGGTACC |
| sh-YOD1-1R | AATTGGTACCAAAAAACAGAAAGGATTAACTGGACAACGTCTTGAATTGTCCAGTTAATCCTTTCTG |
| sh-YOD1-2F | CCGG CCAGAAGTTCACCTGCATTTATTCAAGACGTAAATGCAGGTGAACTTCTGGTTTTTTGGTACC |
| Sh-YOD1-1R | AATTGGTACCAAAAAACCAGAAGTTCACCTGCATTTACGTCTTGAATAAATGCAGGTGAACTTCTGG |
| YOD1-F | AGACGCCTCATAGCACAAA |
| YOD1-R | CTCCTCCCCAAGTGTCATC |
| GAPDH-F | CCTTCCGTGTCCCCACT |
| GAPDH-R | GCCTGCTTCACCACCTTC |
| YOD1 T-UBXL-F | GTGGGCAGCCGGACCGACACGAGTTCACCTGCATTTACTAAA |
| YOD1 T-UBXL-R | CGTGTCGGTCCGGCTGCCCAC |
| YOD1 T-OTUD-F | AGTTACGTCAGGGAAACTTTGCGCTTCACCCTGAGATGCATG |
| YOD1 T-OTUD-R | CAAAGTTTCCCTGACGTAACT |
| YOD1 T-ZnF-F | ACTGATGTCAACCGCTTCACCACCAACTTTGGAGAAGTG |
| YOD1 T-ZnF-R | GGTGAAGCGGTTGACATCAGT |

Supplementary table 2: Clinical Data for a Series of 44 Patients with NTNBC and 32 patients with TNBC.

|  | No | Age | YOD1 expression score | TNM stage | Follow-up time | Status |
| --- | --- | --- | --- | --- | --- | --- |
| NTNBC | 1 | 42 | 1 | I | 76 | NED |
|  | 2 | 45 | 1 | I | 67 | NED |
|  | 3 | 53 | 1 | I | 87 | AWD |
|  | 4 | 51 | 1 | I | 45 | NED |
|  | 5 | 48 | 2 | I | 36 | AWD |
|  | 6 | 47 | 3 | II | 63 | DOD |
|  | 7 | 39 | 2 | II | 85 | NED |
|  | 8 | 62 | 3 | III | 45 | NED |
|  | 9 | 65 | 1 | II | 36 | NED |
|  | 10 | 48 | 2 | I | 32 | NED |
|  | 11 | 53 | 2 | I | 43 | NED |
|  | 12 | 47 | 2 | I | 47 | AWD |
|  | 13 | 69 | 2 | II | 58 | NED |
|  | 14 | 68 | 2 | I | 64 | AWD |
|  | 15 | 42 | 2 | II | 68 | DOD |
|  | 16 | 42 | 3 | III | 54 | AWD |
|  | 17 | 53 | 1 | IV | 58 | NED |
|  | 18 | 52 | 3 | III | 48 | DOD |
|  | 19 | 43 | 2 | IV | 73 | DOD |
|  | 20 | 51 | 2 | I | 37 | NED |
|  | 21 | 51 | 2 | II | 38 | AWD |
|  | 22 | 56 | 2 | IV | 47 | AWD |
|  | 23 | 45 | 1 | III | 47 | AWD |
|  | 24 | 61 | 3 | IV | 53 | AWD |
|  | 25 | 58 | 1 | II | 57 | DOD |
|  | 26 | 47 | 1 | I | 58 | NED |
|  | 27 | 48 | 1 | I | 60 | AWD |
|  | 28 | 43 | 2 | I | 54 | NED |
|  | 29 | 53 | 2 | I | 50 | AWD |
|  | 30 | 48 | 2 | I | 110 | DOD |
|  | 31 | 47 | 2 | I | 80 | AWD |
|  | 32 | 49 | 1 | II | 72 | NED |
|  | 33 | 62 | 1 | II | 60 | NED |
|  | 34 | 64 | 1 | II | 72 | NED |
|  | 35 | 43 | 3 | I | 69 | NED |
|  | 36 | 68 | 3 | II | 70 | DOD |
|  | 37 | 47 | 2 | II | 83 | NED |
|  | 38 | 46 | 2 | II | 58 | DOD |
|  | 39 | 49 | 1 | II | 69 | AWD |
|  | 40 | 54 | 1 | II | 68 | AWD |
|  | 41 | 53 | 3 | I | 74 | NED |
|  | 42 | 52 | 3 | I | 65 | AWD |
|  | 43 | 54 | 3 | I | 48 | DOD |
|  | 44 | 47 | 2 | I | 66 | AWD |
| TNBC | 1 | 42 | 3 | III | 54 | NED |
|  | 2 | 53 | 3 | III | 48 | AWD |
|  | 3 | 52 | 3 | III | 76 | DOD |
|  | 4 | 52 | 3 | III | 46 | NED |
|  | 5 | 51 | 3 | IV | 53 | AWD |
|  | 6 | 51 | 3 | IV | 46 | DOD |
|  | 7 | 56 | 3 | IV | 78 | AWD |
|  | 8 | 62 | 2 | III | 78 | DOD |
|  | 9 | 42 | 2 | IV | 72 | AWD |
|  | 10 | 58 | 2 | III | 77 | NED |
|  | 11 | 42 | 3 | IV | 65 | AWD |
|  | 12 | 58 | 3 | IV | 35 | DOD |
|  | 13 | 43 | 3 | III | 69 | AWD |
|  | 14 | 53 | 3 | III | 74 | NED |
|  | 15 | 43 | 2 | II | 64 | DOD |
|  | 16 | 64 | 2 | II | 42 | DOD |
|  | 17 | 49 | 1 | II | 68 | DOD |
|  | 18 | 62 | 3 | III | 74 | AWD |
|  | 19 | 34 | 3 | II | 78 | NED |
|  | 20 | 43 | 3 | IV | 48 | DOD |
|  | 21 | 43 | 3 | I | 42 | DOD |
|  | 22 | 46 | 3 | IV | 82 | AWD |
|  | 23 | 38 | 3 | IV | 66 | DOD |
|  | 24 | 43 | 3 | III | 64 | AWD |
|  | 25 | 42 | 3 | II | 38 | DOD |
|  | 26 | 56 | 3 | I | 68 | AWD |
|  | 27 | 53 | 3 | II | 28 | DOD |
|  | 28 | 42 | 3 | II | 42 | DOD |
|  | 29 | 43 | 3 | II | 71 | AWD |
|  | 30 | 43 | 2 | IV | 68 | NED |
|  | 31 | 37 | 2 | III | 68 | AWD |
|  | 32 | 47 | 1 | IV | 48 | DOD |
